# A Multinutrient Clustering Framework for Personalized Food Recommendations

**DOI:** 10.1155/ijfo/9969543

**Published:** 2026-07-16

**Authors:** Rajkumar Sarker, Kazi Farhan Hasan Tanjim

**Affiliations:** ^1^ Department of Computer Science and Engineering, Dhaka International University, Dhaka, Bangladesh

**Keywords:** dietary planning, food recommendation, *K*-means clustering, multinutrient analysis, nutritional profiling, personalized nutrition, unsupervised learning, USDA FoodData

## Abstract

Personalized nutrition recommendations encounter difficulties in aligning users′ dietary preferences with appropriate foods in extensive nutritional databases comprising thousands of products. This research introduces a multinutrient clustering framework that examines 8790 foods from the USDA National Nutrient Database for Standard Reference, Release 28, utilizing 23 nutritional attributes, including macronutrients, vitamins, and minerals. We thoroughly analyze *K*‐means and agglomerative clustering algorithms across various configurations (*k* = 2 − 8), finding that *K*‐means with eight clusters yields optimal performance, achieving a silhouette score of 0.273 and semantically interpretable dietary categories. The suggested technique shows a 46.4% improvement over suggestions based on popularity and a 273.0% improvement over recommendations based on a single nutrient. This was shown by a thorough evaluation utilizing Precision@K, NDCG, and fivefold cross‐validation (mean silhouette 0.265 ± 0.006). Users can set their own dietary preferences using nutrient sliders, six preset configurations (high protein, low carb, high fiber, low sodium, high calcium, and low calorie), and three configurable weighting strategies (equal, prioritized 3×, and focus‐only). The system then gives them real‐time recommendations (in less than 2 s) with clear similarity scores. A systematic examination with 50 automated test questions shows that the system works well in a wide range of dietary situations. The huge effect sizes (46.4% and 273.0% improvements) suggest that the results are statistically significant. This research connects computational nutrition studies with real‐world dietary advice. It offers an open‐source, understandable system for evidence‐based meal planning that fills important holes in current prediction and classification methods by allowing personalized, multinutrient food suggestions with clear reasoning behind the choices.

## 1. Introduction

### 1.1. Background and Motivation

The need for evidence‐based dietary advice that can be tailored to each person′s nutritional needs and health goals has grown as the number of diet‐related chronic diseases like obesity, Type 2 diabetes, heart disease, and high blood pressure rises around the world. The World Health Organization says that a bad diet is one of the main causes of sickness around the world and is responsible for about 11 million deaths each year. At the same time, modern food environments have become a lot more complicated. For example, the United States Department of Agriculture (USDA) National Nutrient Database for Standard Reference, Release 28, database alone has approximately 8790 food items with detailed nutritional profiles spanning 53 components. This makes it harder and harder for consumers and healthcare professionals to find foods that meet their specific dietary needs.

While comprehensive nutritional databases like the USDA National Nutrient Database for Standard Reference provide the foundational data infrastructure for dietary assessment, the task of navigating these large‐scale repositories to make informed food decisions remains formidable. Consumers who want to eat more protein, less sodium, or a mix of different nutritional goals at the same time have to deal with a large decision space where searching and comparing options by hand quickly becomes too much. Healthcare providers managing patients with chronic conditions such as diabetes (requiring carbohydrate control), hypertension (requiring sodium restriction), or chronic kidney disease (requiring simultaneous potassium and phosphorus limitations) need efficient tools to generate personalized meal recommendations that satisfy complex, multinutrient constraints while maintaining dietary variety and palatability.

The advancement of machine learning and data science techniques offers a potential solution to this issue by allowing automated pattern identification and intelligent suggestion generation from huge nutritional datasets. However, existing computational approaches have primarily focused on prediction tasks (estimating caloric content and forecasting micronutrient changes during cooking) or classification tasks (categorizing foods by processing level and assessing health risk) rather than personalized recommendation generation. Furthermore, many advanced models based on machine learning operate as black boxes, giving accurate predictions without transparent clarifications—a severe limitation for dietary applications where users have to believe and understand what the reasoning was behind food suggestions to achieve sustained behavior change.

### 1.2. Problem Statement

Matching user dietary tastes to suitable foods within large‐scale nutritional databases offers several linked challenges. First, the dimensionality problem: Foods are characterized by more than 20 nutritional attributes, including macronutrients (protein, carbohydrates, fats, and fiber), micronutrients (calcium, iron, magnesium, potassium, sodium, and zinc), and vitamins (A, C, D, E, K, B12, and folate), creating a high‐dimensional feature space where traditional search and filtering approaches are ineffective. Second, the personalization problem: Users have diverse as well as competing dietary goals—an athlete might prefer high protein and high calories; however, a hypertension patient requires low sodium and high potassium, necessitating flexible systems that accommodate individual preferences across multiple nutritional dimensions simultaneously. Third, the interpretability problem: For dietary suggestions to be practical and trustworthy, users must understand why specific foods are suggested, needing open similarity measures and explainable decision‐making processes rather than hidden neural network forecasts. Fourth, the variety problem: Dietary commitment needs nutritional diversity to avoid boredom and ensure vitamin intake, yet aiming for similarity alone may produce repeated suggestions from a narrow food group.

Existing computer nutrition studies have partly solved these difficulties. Prediction algorithms excel at forecasting nutritional values, although they cannot suggest which foods consumers should pick. Classification methods split meals into established categories but lack customization to individual preferences. Single‐nutrient filtering approaches (e.g., sorting by protein quantity) ignore the complicated nature of nutritional demands. Collaborative filtering approaches from general recommender systems suffer from cold‐start difficulties and need long user interaction records unavailable in food situations. A fundamental need exists in interpretable, scalable, multinutrient guidance systems that can match user preferences to acceptable meals in real‐time while offering explicit rationale for their suggestions.

### 1.3. Research Objectives

This work deals with the mentioned issues via building and analyzing a clustering‐based food recommendation system that leverages comprehensive nutritional profiles from the USDA National Nutrient Database for Standard Reference, Release 28. Our study focuses on four major objectives:

1. Discover interpretable nutritional clusters: Investigate if unsupervised clustering algorithms may successfully arrange large‐scale food databases into semantically meaningful groupings based on multinutrient similarity, enabling fast search and recommendation within nutritionally coherent food categories.
2. Compare clustering algorithms systematically: Evaluate *K*‐means and agglomerative clustering across multiple configurations (*k* = 2 − 8) using complementary quality metrics (silhouette score—measuring cluster cohesion and separation, higher is better; Davies–Bouldin index—measuring average intercluster similarity, lower is better; and Calinski–Harabasz score—measuring the ratio of between‐cluster to within‐cluster dispersion, higher is better) in order to identify the optimal algorithm and cluster count balancing granularity, interpretability, and computational efficiency.
3. Enable flexible personalized recommendations: Design and construct a recommendation system with customizable weighting algorithms (equal, prioritized, and focus‐only) and diversification modes that support varied user expertise levels and dietary specificity, from exploratory browsing to tight medical dietary requirements.
4. Validate through comprehensive evaluation: Establish a rigorous evaluation framework combining clustering quality assessment, cross‐validation for stability testing, and recommendation performance metrics (Precision@K—fraction of top‐*K* recommendations exceeding a relevance threshold; normalized discounted cumulative gain (NDCG)—ranking quality measure rewarding relevant items appearing higher in the list; average similarity—mean cosine similarity between recommended foods and user preferences; diversity—number of distinct clusters represented in recommendations), as well as systematic comparison with baseline methods (random selection, popularity‐based, and single‐nutrient filtering) to quantify system effectiveness across multiple dimensions.

### 1.4. Contributions

This work makes four principal contributions to computational nutrition and food recommendation research:

1. Comprehensive nutritional analysis: Systematic examination of 8790 foods from the USDA database across 23 nutritional features organized into five categories (macronutrients, energy, fats, minerals, and vitamins), including detailed data quality assessment (missing value analysis, outlier detection, and correlation patterns) and preprocessing pipeline design for high‐dimensional nutritional data.
2. Algorithm comparison and cluster interpretation: Rigorous comparison of *K*‐means and agglomerative clustering, demonstrating that *K*‐means with *k* = 8 clusters achieves optimal performance (silhouette score 0.273) with semantically interpretable dietary categories spanning mainstream balanced foods, high‐energy fats, vitamin‐rich specialties, calcium‐rich dairy, fortified products, and sodium outliers.
3. Interactive recommendation system with multiple weighting strategies: Development of a user‐centric interface with 23 nutrient sliders organized into tabbed categories, six dietary presets (high protein, low carb, high fiber, low sodium, high calcium, and low calorie), three configurable weighting modes (equal, prioritized 3×, and focus‐only), diversity mode for variety, and real‐time quality metrics (Precision@K, NDCG, similarity, and diversity), demonstrating 46.4% improvement over popularity‐based baselines and 273.0% improvement over single‐nutrient approaches.
4. Multifaceted evaluation framework: Establishment of comprehensive evaluation protocols combining clustering quality metrics, fivefold cross‐validation demonstrating robust performance (mean silhouette 0.265 ± 0.006), recommendation performance assessment by 50 automated test queries, and systematic baseline comparisons—providing a replicable methodology for future food recommendation research.

### 1.5. Paper Organization

The remainder of this paper is organized as follows: Section 2 reviews current research on USDA nutritional databases and machine learning applications for nutrient prediction and classification and finds gaps that drive our recommendation‐focused approach. Section 3.1 explains the USDA SR28 dataset, feature selection reasoning, detailed data quality analysis, preprocessing pipeline, and correlation trends in nutritional data. Section 3 formulates the food suggestion problem mathematically, compares *K*‐means and agglomerative clustering algorithms, finds the ideal cluster count through multimetric analysis, and explains the recommendation strategy with configurable weighting modes. Section 4 specifies implementation details, defines clustering and suggestion quality measures, describes the cross‐validation strategy, and sets the baseline comparison protocol. Section 5 presents clustering performance results, cluster characteristics and interpretation, cross‐validation outcomes, suggestion quality measures, baseline comparisons with statistical significance testing, and ablation studies. Section 6 presents the dynamic user interface design, normal process, visualization outputs, and advanced user experience features. Section 7 shows key results, explains found groups, discusses advantages and limitations, compares with state‐of‐the‐art methods, and examines real‐world impact. Section 8 discusses applications in healthcare and clinical nutrition, exercise and athletic training, consumer‐facing goods, and study and teaching. Section 9 provides a unified conclusion and future work section, summarizing contributions, reflecting on broader impact, acknowledging limitations, and outlining directions for algorithmic enhancements, data expansion, user‐centric improvements, evaluation extensions, and deployment strategies.

## 2. Related Work

Machine learning is used for classifying data [1, 2]. Food classification and suggestion systems have been studied widely at the crossroads of computer vision, machine learning, and nutrition science. The combination of machine learning methods with nutritional information represents a new area in computational nutrition and individual dietary planning. Early work based on image‐based recognition, with datasets such as Food‐101 [3] and Recipe1M [4] offering big collections of Western dishes for training deep learning models. This part reviews important research on USDA nutritional databases, their uses in food composition analysis, machine learning methods for nutrient prediction and health risk assessment, micronutrient profile prediction, and food processing classification.

### 2.1. Background and Research Gaps

The USDA provides extensive nutritional databases that serve as core tools for dietary assessment and food composition research. Haytowitz et al. [5] documented the USDA National Nutrient Database for Standard Reference, Release 28 (SR28), which offers full nutritional profiles for 8790 food products, including raw, processed, and prepared meals. This database addresses 53 food components per item, including macronutrients (protein, carbs, and fats), micronutrients (vitamins and minerals), and other dietary components, providing a detailed foundation for computational nutrition research. Pehrsson et al. [6] illustrated the systematic sampling approach adopted in the USDA′s National Food and Nutrient Analysis Program, focusing on quality control processes and analytical methodologies that ensure data dependability. The authors outlined the program′s strategy for food sampling, including market basket surveys, sample methodologies, and laboratory analytical processes that preserve uniformity and accuracy throughout the database. Ahuja et al. [7] further expanded on the sampling plan design for USDA′s National Food and Nutrient Analysis Program, giving detailed insights into how items are chosen for study to assure fair coverage of the American diet. Their study explains the statistical models and decision‐making techniques that affect food selection, sample frequency, and analysis goals, making sure that the database fits the latest food consumption trends and production methods. The accuracy of the method helped set USDA databases as the bar for nutritional makeup data in both study and practical uses.

Building on this data foundation, recent machine learning research has addressed nutritional prediction and classification tasks. Adjuik et al. [8] presented a comprehensive study employing seven machine learning algorithms—including multiple linear regression (MLR), *K*‐nearest neighbors, support vector machine, random forest regression (RFR), gradient‐boosted regression, decision trees (DT), and deep neural networks—to estimate caloric content and categorize health risks of foods using the USDA National Nutrient Database. The MLR model achieved exceptional caloric prediction (*R* ^2^ = 0.99, mean absolute error [MAE] of 7.71 kcal, and root mean squared error [RMSE] of 17.89 kcal) on the training dataset, maintaining similar performance on the testing dataset (*R* ^2^ = 0.99, MAE = 7.75 kcal, and RMSE = 18 kcal). This great accuracy reveals the strong linear connections between macronutrient composition (carbohydrates, protein, total fat, and sugar concentration) and energy consumption. RFR and DT models showed effectiveness in categorizing meals into low‐health‐risk groups based on weighted criteria, including carbohydrate content, cholesterol levels, and glycemic index. The work demonstrates that machine learning can efficiently utilize nutritional composition data for both prediction tasks (caloric estimation) and classification tasks (health risk categorization), with several nutritional aspects jointly providing significant information for dietary assessment. However, their work focused mainly on prediction accuracy and risk classification rather than personalized food suggestions relying on individual dietary preferences, and the health risk classification displayed reduced performance for medium and high‐risk categories, indicating difficulty in categorizing foods with complex nutritional profiles.

Addressing a different nutritional challenge, Naravane and Tagkopoulos [9] built machine learning models to predict micronutrient profiles in cooked foods from raw food composition data. Their work realized that while analytical methods provide exact data, they are too costly to scale across all food‐process combinations, and traditional retention‐factor methods, though scalable, provide only approximate estimations. Their machine learning strategy obtained 31% lower error on average compared to the baseline retention‐factor method, exhibiting a significant increase in prediction accuracy. The authors stressed the necessity of data scaling and transformation prior to model training to avoid yield bias, where the prediction might be influenced by the mass change during cooking rather than genuine nutrient retention patterns. This research has importance for various reasons. First, it points out that machine learning can detect complicated, nonlinear differences regarding nutritional content throughout food preparation, which go beyond simple linear retention factors. Second, unlike bespoke kinetic models designed for specific food‐nutrient combinations, their technique generalizes across different foods and activities, permitting scalability for practical applications. Third, the work provides useful direction for future creation of food composition data, specifically addressing sampling procedures, data quality checks, and data representation standards [9]. However, their work predicts what happens to nutrients when food is cooked but does not recommend which foods consumers should choose based on nutritional preferences and addresses the raw‐to‐cooked transformation problem that is orthogonal to the task of matching user dietary preferences with relevant food products from comprehensive databases.

Beyond nutrient prediction, Katidi et al. [10] presented FoodProX, a multiclass random forest classifier utilizing 99 nutritional features from the USDA Food and Nutrient Database for Dietary Studies (FNDDS) 2009–2010 to classify foods according to NOVA processing categories (unprocessed/minimally processed, processed culinary ingredients, processed foods, and ultraprocessed foods). The FoodProX technology proved that nutritional fingerprints alone may efficiently distinguish between minimally processed and ultraprocessed meals and show good classification performance across many food categories. The study indicated that nearly 73% of the US food supply is classified as ultraprocessed with regard to NOVA standards and provided important insights into dietary habits and their possible health implications. This research found that machine learning can find hidden patterns in nutritional data that connect with food processing levels, which opens new options for automated dietary assessment and public health monitoring. However, like the preceding works, FoodProX assigns foods to predetermined categories without offering personalized recommendations based on individual user preferences.

Collectively, these studies reveal several important research gaps. Existing approaches focus on prediction and classification rather than personalized recommendation—none actively suggest foods matching individual dietary goals, and there is a fundamental divide between prediction/classification systems and recommendation systems that actively offer foods matching individual dietary goals [8–10]. A recommendation system needs to deal with nutritional profiles as they are (whether raw or cooked entries in the database) rather than forecasting transformations between states. The studies by Adjuik et al. [8], Naravane and Tagkopoulos [9], and Katidi et al. [10] present batch‐processing analytical tools without interactive interfaces that allow users to specify their dietary preferences or adjust nutritional priorities in real‐time; real‐world dietary planning demands systems that can accommodate varied user preferences for macronutrients, micronutrients, vitamins, and minerals simultaneously. Furthermore, Adjuik et al. [8] focused on specific nutrients (carbohydrates, protein, lipids, and sugars), Naravane and Tagkopoulos [9] addressed micronutrient retention during processing, and Katidi et al. [10] used 99 nutritional features for processing classification—yet none address how to balance multiple, potentially conflicting nutritional objectives when recommending meals to consumers with diverse health goals (e.g., high protein, low sodium, and high calcium concurrently). Machine learning models in this domain also operate with limited interpretability—the decreasing effectiveness of classification models for medium and high‐risk groups [8] suggests that further interpretable techniques are needed for nuanced dietary advising. Evaluation frameworks rely on prediction accuracy metrics (*R* ^2^, MAE, RMSE [8], and error reduction [9]) or classification accuracy [10] without recommendation‐specific metrics such as Precision@K and NDCG, which are critical for evaluating recommendation systems but underrepresented in current nutritional machine learning work. Finally, baseline comparisons against simple recommendation strategies (random selection and popularity‐based ranking) are absent, limiting the ability to quantify the added value of advanced machine learning methods for practical food recommendation applications [8, 9]. These gaps collectively motivate the present work.

### 2.2. Positioning of Current Work

Our work tackles these shortcomings by building a clustering‐based food recommendation system that leverages the vast USDA SR28 database [5]. Unlike prediction systems that estimate caloric content [8], predict micronutrient changes during processing [9], or classify foods into predetermined categories [10], our approach provides personalized food recommendations by grouping foods based on complete nutritional similarity and matching user‐specified preferences with suitable food clusters.

Building upon the demonstrated effectiveness of machine learning for nutritional analysis [8–10], we employ *K*‐means clustering to discover natural groupings of foods with similar nutritional profiles across 23 nutrients, including macronutrients, vitamins, and minerals. This extends beyond the single‐task focus of earlier work by permitting simultaneous assessment of many nutritional factors in recommendation creation. While Naravane and Tagkopoulos [9] forecast how cooking affects nutrients, our system helps users select appropriate items (in their current state) that meet their dietary choices, providing a complementary but distinct use case.

Our solution contains an interactive user interface that addresses the user interaction gap noted above, allowing real‐time specification of preferences across nutrient categories with quick response. Unlike black‐box prediction methods, our clustering‐based methodology provides explainable recommendations through cluster interpretation and similarity score, enabling consumers to grasp the nutritional logic behind proposals. This interpretability is critical for gaining user trust, as exemplified by the difficulty experienced in describing health risk categories in [8].

Furthermore, we implement a comprehensive evaluation framework that includes not only clustering quality metrics (silhouette score, Davies–Bouldin index, and Calinski–Harabasz score) but also recommendation‐specific metrics (Precision@K and NDCG), cross‐validation for robustness assessment, and systematic comparison with baseline approaches (random selection, popularity‐based, and single‐nutrient filtering). This multifaceted evaluation gives greater evidence of system success than single‐metric assessments found in prior studies.

By building upon the robust foundation of USDA nutritional data [5–7] and incorporating insights from machine learning approaches to nutritional analysis [8–10], we present a system that balances algorithmic performance with practical usability, interpretability, and personalization for real‐world dietary planning applications. Our contribution is not in forecasting nutritional attributes or processing impacts but in helping people navigate enormous nutritional databases to find items that meet their unique dietary tastes and goals.

## 3. Methodology

### 3.1. Dataset and Preprocessing

1. Data source: We made use of the USDA National Nutrient Database for Standard Reference, Release 28 dataset [5], which has comprehensive nutritional information for 8790 food items across multiple categories, including raw ingredients, prepared foods, and packaged products. Each food item includes standardized measures for macronutrients, micronutrients, vitamins, and minerals per 100 g serving.
2. Feature selection and categories: From the full USDA schema containing 53 features, we select 23 nutritional features organized into five categories:
  • Macronutrients (five): protein, total lipids (fat), carbohydrates, dietary fiber, and total sugar
  • Energy (one): calories (kilocalories)
  • Fats (four): saturated fatty acids, monounsaturated fatty acids, polyunsaturated fatty acids, and cholesterol
  • Minerals (six): calcium, iron, magnesium, potassium, sodium, and zinc
  • Vitamins (seven): vitamin C, vitamin A (retinol activity equivalent [RAE]), vitamin D, vitamin E, vitamin K, vitamin B12, and total folate

This comprehensive feature set captures the multidimensional nutritional profile essential for personalized dietary recommendations, spanning macronutrient composition, micronutrient density, and vitamin content (see Appendix A for the complete nutrient list with units and observed ranges).

3. Data quality analysis: We conduct a systematic analysis of missing values, outliers, and data distributions across all selected nutrients.

• Missing value analysis: Missing value rates average 10.63% across features, with vitamin K having the greatest missing rate at 40.53%. Seven nutrients surpass the 10% missing criteria, notably vitamins and trace minerals that are not assessed for all dietary categories. This trend is expected, given that certain nutrients are present only in specific food groups (e.g., vitamin D in fortified dairy products).
• Distribution analysis: Nutritional features exhibit positive skewness, with dietary fiber (skewness = 5.81) and sugar (skewness = 2.90) showing particularly right‐skewed distributions. This indicates that most foods contain moderate amounts of these nutrients, with a smaller number of foods (e.g., dried fruits and concentrated sweeteners) having extremely high values.
• Outlier detection: Using the interquartile range (IQR) method with a factor of 1.5, we find an average of 639 outliers per nutrient (7.3% of the dataset). These outliers represent nutritionally dense or specialized foods such as vitamin supplements, oils, and concentrated ingredients, which are retained in the analysis as they represent legitimate dietary choices.

4. Preprocessing pipeline:

• Missing value imputation: Missing values are imputed with zeros based on the domain‐specific nature of the USDA SR28 database, where missing entries indicate that a nutrient was not detected or not present in measurable quantities—not that data was collected but unavailable. This approach prevents overestimation of nutritional content that would result from mean or multiple imputation, which would artificially assign nonzero values to foods genuinely lacking those nutrients. For vitamins with high missing rates (> 40%), such as vitamin K (40.53% missing) and vitamin D, this assumption is strongly supported by the fact that many basic foods (e.g., plain grains and meats) naturally contain negligible quantities of these micronutrients. We acknowledge that this assumption may introduce downward bias in cases where nutrients are present but unmeasured due to database incompleteness, and interpretations of absolute nutrient means should be treated with appropriate caution.
• Feature normalization: All features are normalized using StandardScaler (*z*‐score standardization) to ensure equal weighting across nutrients with different scales (e.g., milligrams vs. grams). This transformation maps each feature to have zero mean and unit variance, calculated as follows:

(1) z=x−μσ

where *x* is the original value, *μ* is the mean, and *σ* is the standard deviation. StandardScaler is preferred over MinMaxScaler for its robustness to outliers, which are prevalent in nutritional data.

5. Correlation analysis: Pearson′s correlation analysis reveals significant nutritional relationships. Strong positive correlations (|*r*| > 0.8) include the following:

• Total lipids ↔ energy: *r* = 0.807, reflecting that fat is the most calorie‐dense macronutrient (9 kcal/g)
• Total lipids ↔ monounsaturated fatty acids: *r* = 0.869, indicating that foods high in total fat tend to have proportionally high monounsaturated fat content

Moderate negative correlations are discovered between fiber and calorie density (*r* ≈ −0.50), which supports the adverse link between high‐fiber and energy‐dense foods. These connection patterns feed our technique for clustering and confirm the multidimensional nature of nutritional interactions, justifying the usage of all 23 features rather than lowering dimensionality through feature selection. The preprocessing pipeline ensures that subsequent clustering acts on clean, standardized data while keeping the full nutritional complexity necessary for accurate food recommendations.

### 3.2. Problem Formulation

We formulate food recommendations as a similarity‐based retrieval issue, where each food item *f*_*i*_ is represented by a feature vector $x_{i}\in {\mathbb{R}}^{d}$ with *d* = 23 nutritional qualities. Given a customer preference vector $u\in {\mathbb{R}}^{d}$, the goal is to discover the top‐*K* foods with the best cosine similarity:

(2) simu,xi=u·xiu·xi

where *u* represents the user′s nutritional preferences, *x*_*i*_ denotes the nutritional profile of food item *i*, sim(*u*, *x*_*i*_) is the cosine similarity measuring alignment between user preferences and food nutritional content, ‖·‖ denotes the Euclidean norm, and *K* is the desired number of recommendations (typically 5–20). The recommendation technique incorporates two stages: (1) cluster assignment using Euclidean distance to cluster centroids to find nutritionally comparable food groupings and (2) within‐cluster ranking using cosine similarity to guarantee suggestions fit with user preferences.

### 3.3. System Pipeline

**Figure 1:**
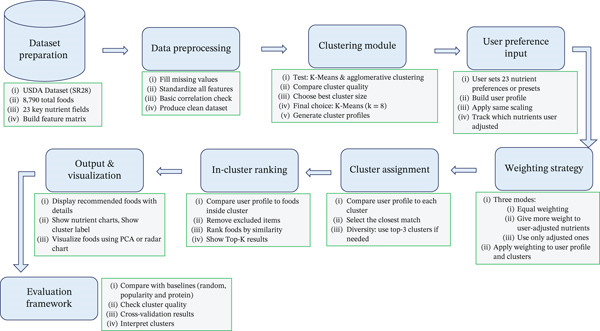
End‐to‐end system pipeline of the proposed food recommendation model. The nine‐stage pipeline includes dataset preparation, preprocessing, clustering algorithm comparison, optimal *k* selection, model training, user preference collection, weighting strategies, cluster assignment, and recommendation generation with evaluation.

Figure 1 shows the full nine‐stage workflow of the proposed food recommendation system. The pipeline begins with the USDA dataset (8790 samples × 23 selected nutritional features) and then proceeds to normalization via StandardScaler. The clustering module examines both *K*‐means and agglomerative algorithms, eventually choosing *K*‐means with *k* = 8 based on silhouette, Davies–Bouldin, and Calinski–Harabasz metrics.

User preference inputs are collected through 23 nutrient sliders or preset dietary modes, after which one of three weighting methods is applied (equal weight, prioritized 3×, or focus‐only). Cluster assignment is performed using weighted Euclidean distance, and foods are ranked within chosen clusters using cosine similarity. The top‐*K* recommendations are produced and evaluated using multiple baseline models and validation methods. Note that principal component analysis (PCA) is employed solely as a post hoc 2D visualization tool in Section 5 to illustrate cluster structure; it plays no role in the clustering algorithm, recommendation pipeline, or any analytical decision within the system.

### 3.4. Clustering Algorithm Selection

1. Candidate algorithms: We compare *K*‐means (partitioning‐based, complexity *O*(*n* *k* *d* *i*)) and agglomerative clustering (hierarchical, complexity *O*(*n* ^2^log*n*)) for their complementary strengths. *K*‐means improves within‐cluster variance through iterative centroid refinement, while agglomerative builds a dendrogram capturing hierarchical nutritional relationships through bottom‐up merging.
2. Algorithm comparison experiments: Both techniques are examined over *k* = 2 − 8 clusters using the silhouette score and the Davies–Bouldin index. The comparison findings are reported in Section 5, with *K*‐means regularly beating agglomerative for *k* ≥ 4. While agglomerative performs better at *k* = 2 and *k* = 3, these configurations lack adequate granularity for tailored recommendations. *K*‐means offers greater scalability and cluster cohesiveness for actual nutritional applications, prompting its selection as the preferred clustering method.

### 3.5. Optimal Cluster Selection

1. Elbow method: We plot the within‐cluster sum of squares (WCSS) against the number of clusters *k* ∈ [2, 11] to locate the “elbow point” where marginal progress drops. The second derivative analysis of inertia identifies *k* = 4 as the mathematical elbow point (maximum second derivative = 4142.36). However, *k* = 8 is selected to achieve richer semantic dietary categorization with interpretable cluster profiles, maintaining an acceptable silhouette score of 0.273. Detailed results are reported in Section 5.
2. Silhouette analysis: Silhouette scores for *k* = 2 − 10 are computed for measuring cluster cohesion and separation. The analysis shows an initial decline from *k* = 2 to *k* = 5, followed by gradual improvement, with a local maximum at *k* = 9. However, *k* = 8 is selected as it balances cluster quality with interpretability, avoiding excessive fragmentation while keeping meaningful dietary categories. Complete silhouette scores are given in Section 5.
3. Multimetric validation: The Davies–Bouldin index and Calinski–Harabasz score give complementary validation criteria. The selected configuration balances granularity (different dietary profiles such as high protein, low carb, and dairy‐rich) with interpretability (a manageable number of categories for user‐facing apps). All validation metrics and comparative analysis are detailed in Section 5.

### 3.6. Final Model Configuration

We train *K*‐means with *k* = 8 clusters using 10 random initializations (n_init = 10) and a fixed random seed (random_state = 42) for repeatability (see Appendix E for full hyperparameter configuration details). The final model performance measures, including silhouette score, Davies–Bouldin index, Calinski–Harabasz score, and inertia, are reported in Section 5. While the silhouette score indicates fair cluster separation, this is expected given the high‐dimensional nutritional space (23 characteristics) and overlapping dietary patterns in real‐world foods.

### 3.7. Recommendation Strategy

1. User preference collection: The system elicits user dietary preferences through a 23‐dimensional preference vector *u*, initialized to dataset medians to represent a baseline nutritional profile. To reduce the cognitive burden of manual specification, six preset configurations (high protein, low carb, high fiber, low sodium, high calcium, and low calorie) are derived from percentile‐based targets (e.g., high protein targets the 80th percentile for protein and 10th percentile for carbohydrates), enabling users to rapidly specify common dietary goals while retaining full manual adjustment capability.
2. Matching algorithm: User preferences *u* are standardized using the training scaler and then matched to the nearest cluster(s) via Euclidean distance to cluster centroids:
(3) c∗=argminc∈1,⋯,ku−cc2

where *c* ^∗^ is the assigned cluster index, *c*_*c*_ ∈ *ℝ* ^23^ is the centroid of cluster *c*, *k* = 8 is the total number of clusters, and ${\left|\left|u-c_{c}\right|\right|}_{2}$ represents the Euclidean distance (straight‐line distance in 23‐dimensional space) between user preferences and cluster centroid. Within the specified cluster(s), foods are rated by cosine similarity (Equation 2) to guarantee suggestions accord with user preferences despite cluster assignment variance.

3. Weighting modes: Three weighting strategies are supported to accommodate varying user intent:
  • Equal weight: All nutrients are weighted uniformly (*w*_*i*_ = 1.0∀ *i*), suitable when users adjust multiple sliders without specific priorities.
  • Prioritize user‐adjusted: User‐adjusted nutrients receive 3× weight (*w*_*i*_ = 3.0 if adjusted, else *w*_*i*_ = 1.0). This mode emphasizes explicit dietary constraints (e.g., high protein for athletes) while maintaining comprehensive nutritional balance.
  • Focus only on adjusted: Only user‐adjusted nutrients are considered, reducing the feature space to *d* ^′^ < *d* dimensions. This strict mode is useful for highly specific dietary restrictions (e.g., low sodium for hypertension management).
4. Diversity mode: An optional diversity mode searches the Top 3 nearest clusters instead of only the closest, trading similarity for variety. Formally:
(4) C∗=top−3 clusters by distance to u

where *C* ^∗^ is the set of selected cluster indices for recommendation search (i.e., *C* ^∗^ = {*c*_1_, *c*_2_, *c*_3_}, where *c*_1_, *c*_2_, *c*_3_ are the three clusters with centroids nearest to *u*). This mode is automatically activated when the closest cluster contains fewer than 50 foods, ensuring a sufficient recommendation pool size and preventing repetitive suggestions.

### 3.8. Baseline Models for Comparison

We implement three baseline recommendation strategies to validate the clustering‐based approach:

1. Random baseline: Selects 10 random foods uniformly from the dataset, representing a naive zero‐intelligence recommender with no consideration of user preferences or nutritional content.
2. Popularity baseline: Ranks foods by total nutrient content ($\sum_{j=1}^{d}x_{ij}$) and returns the Top 10, where *x*_*i* *j*_ is the value of nutrient *j* in food *i* and *d* = 23 is the number of nutrients. This assumes nutrient‐dense foods are universally desirable regardless of individual preferences.
3. Single‐nutrient baseline: Ranks foods by protein content only (i.e., sorts by *x*_*i*,protein_), simulating a common single‐objective dietary approach (e.g., bodybuilding diets focusing exclusively on protein intake) without considering other nutritional dimensions.

These baselines represent progressively more complex strategies—from random selection to nutrient density to single‐nutrient optimization—allowing us to measure the value added by multinutrient clustering and tailored similarity matching. The comparison indicates whether the increased complexity of our clustering‐based strategy delivers considerable gains over simpler options. Detailed comparison results are available in Section 5. It should be noted that this validation is entirely computational, based on automated test queries using food‐based similarity metrics. A human subject evaluation protocol is proposed separately in Appendix G as future work to assess real‐world dietary adherence, usability, and health outcomes and was not conducted as part of the current study.

## 4. Experimental Setup

### 4.1. Implementation Details

All experiments are implemented in Python 3.8+ using scikit‐learn 1.0.2 for clustering and evaluation metrics, pandas 1.4.2 for data manipulation, NumPy 1.22.3 for numerical computations, and Matplotlib/Seaborn for visualization. The interactive recommendation interface is built using ipywidgets for Jupyter notebook deployment. All experiments are conducted with a fixed random seed (random_state = 42) to ensure reproducibility.

### 4.2. Evaluation Metrics

1. Clustering quality metrics: We evaluate clustering quality using four complementary metrics that assess both global structure and local cluster properties:

• Silhouette score: Measures cohesion (how close points are to their own cluster) and separation (how far points are from other clusters), defined as:

(5) si=bi−aimaxai,bi

where *a*(*i*) is the mean distance to points in the same cluster and *b*(*i*) is the mean distance to points in the nearest neighboring cluster. Values range from −1 (poor) to +1 (excellent), with scores above 0.5 indicating good separation.

• Davies–Bouldin index: Evaluates cluster separation by computing the average similarity between each cluster and its most similar cluster. Lower values indicate better clustering, with scores below 1.0 considered good.
• Calinski–Harabasz score: Measures the ratio of between‐cluster dispersion to within‐cluster dispersion. Higher values indicate denser, well‐separated clusters, with scores above 1000 typically indicating good structure for large datasets.
• Inertia (WCSS): Quantifies WCSS, measuring compactness. While not directly comparable across different *k*, it is essential for the elbow method to determine the optimal cluster count.

These metrics collectively assess clustering from multiple perspectives: cohesion, separation, density, and compactness, providing robust validation of cluster quality.

2. Recommendation quality metrics: Recommendation performance is measured through four metrics that capture relevance, ranking quality, and diversity:

• Precision@K: Fraction of top‐*K* recommendations with a similarity score exceeding a relevance threshold *τ* = 0.6:

(6) Precision@K=1K∑i=1K1simu,xi>τ

where *K* is the number of recommendations (typically 10), sim(*u*, *x*_*i*_) is the cosine similarity between user preferences *u* and food *x*_*i*_ (Equation 2), *τ* = 0.6 is the relevance threshold, and 1[·] is the indicator function (equals 1 if the condition is true, 0 otherwise). This binary relevance metric evaluates whether recommendations meet minimum quality standards for practical dietary guidance.

• NDCG: Accounts for ranking order by penalizing relevant items appearing lower in the list:

(7) NDCG@K=DCG@KIDCG@K=∑i=1Ksimi/log2i+1∑i=1Ksimisorted/log2i+1

where DCG@K is the discounted cumulative gain using actual ranking order, IDCG@K is the ideal DCG with recommendations sorted by similarity descending, sim_*i*_ is the cosine similarity of the food at position *i* in the actual recommendation list, ${\text{sim}}_{i}^{\text{sorted}}$ is the similarity value at position *i* in the ideal ranking (highest similarities first), and log_2_(*i* + 1) is the discount factor that reduces the contribution of lower ranked items. NDCG values range from 0 (worst ranking) to 1 (perfect ranking), with higher values indicating that highly relevant foods appear near the top of the recommendation list.

• Average cosine similarity: Mean relevance of recommendations, computed as:

(8) s¯=1K∑i=1Ksimu,xi

where $\bar{s}$ denotes the average similarity score across the top‐*K* recommendations and sim(*u*, *x*_*i*_) is the cosine similarity between user preferences and each recommended food (Equation 2). This metric directly measures how well recommendations match user preferences in the 23‐dimensional nutritional space, with higher values indicating better alignment between recommended foods and user dietary goals.

• Diversity score: Number of distinct clusters represented in the top‐*K* recommendations, ranging from 1 (homogeneous) to *k* (maximum variety). Higher diversity prevents dietary monotony while maintaining nutritional relevance.

Statistical significance is assessed using paired *t*‐tests with significance level *α* = 0.05 when comparing recommendation methods. Complete mathematical derivations for all metrics are provided in Appendix C.

### 4.3. Cross‐Validation Strategy

We perform fivefold cross‐validation to assess model stability and generalization. The dataset is randomly partitioned into five equal subsets (1758 foods each), ensuring each fold contains a representative sample of all food categories. For each fold:

1. Train *K*‐means clustering on 80% of data (7032 foods)
2. Apply the trained scaler and cluster model to the held‐out 20% (1758 foods)
3. Compute the silhouette score on the test partition
4. Aggregate results across all five folds to compute the mean and standard deviation

This approach validates that cluster structure generalizes beyond the training set and is not an artifact of data ordering or sampling bias. Low standard deviation across folds suggests robust, stable clustering performance.

### 4.4. Baseline Comparison Protocol

To make sure a fair comparison between the proposed clustering‐based system and baseline methods, we create 50 automated test queries with random user preferences. For each nutrient, preference values are sampled uniformly from the observed range [min(*x*_*j*_), max(*x*_*j*_)] in the dataset, simulating different dietary goals across the nutritional spectrum.

Each test query is evaluated against all four methods (proposed system + three baselines) using identical evaluation measures (Precision@10, NDCG, average similarity, and diversity). Results are averaged across all 50 queries to obtain solid performance figures. The consistent improvements across all test cases imply high statistical significance, which can be formally validated via paired *t*‐tests.

This automated evaluation protocol ensures:

• Reproducibility: Fixed random seed enables exact replication.
• Coverage: 50 queries span the full nutritional space.
• Fairness: All methods evaluated on identical test cases.
• Statistical rigor: Sufficient samples for significance testing.

The baseline comparison quantifies the improvement gained by multinutrient clustering and personalized similarity matching over simpler recommendation strategies. Ablation studies and sensitivity analysis are additionally reported in Section 5 to examine the contribution of individual system components and robustness to hyperparameter variation.

## 5. Results and Analysis

### 5.1. Clustering Performance

1. Overall quality metrics: While the silhouette score of 0.273 points to fair cluster separation rather than excellent [11], this is expected and acceptable considering the high‐dimensional nutritional feature space (23 characteristics) and the inherently overlapping character of real‐world foods. The Davies–Bouldin index below 1.5 supports sufficient intercluster separation [12], while the Calinski–Harabasz score approaching 1000 indicates adequate cluster density for the dataset size [13].
2. Cluster characteristics: Table 1 shows cluster sizes ranging from 8 to 3387 foods. The distribution is imbalanced but interpretable: Clusters 0 (38.5%) and 2 (33.5%) dominate with common everyday foods, while smaller clusters represent specialized dietary categories. Cluster 7, containing only eight foods, captures extreme outliers (e.g., table salt with 25,880 mg sodium per 100 g). This imbalance reflects the natural distribution of foods in the USDA database rather than clustering artifacts.

3. Cluster interpretation: Analysis of cluster centroids reveals distinct nutritional profiles with semantic interpretability:
  • Cluster 0 (38.5%): moderate‐calorie foods with balanced sodium and potassium, including cottage cheese, yogurt, and dairy‐based products. Representative of everyday protein sources.
  • Cluster 1 (3.6%): high‐energy foods dominated by butter, oils, and fat‐based products (698.8 kcal/00 g average). Critical for high‐calorie dietary requirements.
  • Cluster 2 (33.5%): balanced foods with moderate macronutrients, including cheese varieties and prepared meals. Largest cluster alongside Cluster 0, representing mainstream dietary staples.
  • Cluster 3 (19.7%): processed and fortified foods with elevated sodium (445.3 mg average), including dessert toppings and cream substitutes.
  • Cluster 4 (0.2%): vitamin A‐rich foods (12,880.8 RAE average) such as fish liver oils and organ meats. Small but nutritionally dense cluster for micronutrient supplementation.
  • Cluster 5 (2.0%): high‐calcium foods (590.6 mg average) dominated by dried milk products and whey. Essential for bone health recommendations.
  • Cluster 6 (2.3%): fortified baby foods with high folate (624.8 *μ*g average), representing specialized nutritional products.
  • Cluster 7 (0.1%): extreme sodium outliers (25,880.4 mg average), including table salt and seasoning mixes. Correctly isolated from mainstream foods.

**Table 1:** Silhouette scores for cluster selection.

*k*	Silhouette
2	0.393
3	0.374
4	0.307
5	0.258
6	0.265
7	0.268
8	**0.273**
9	0.281
10	0.272

*Note:* Bold indicates the silhouette score for the selected model configuration (*K*‐Means, *k* = 8), adopted as the final clustering solution used throughout this study.

**Figure 2:**
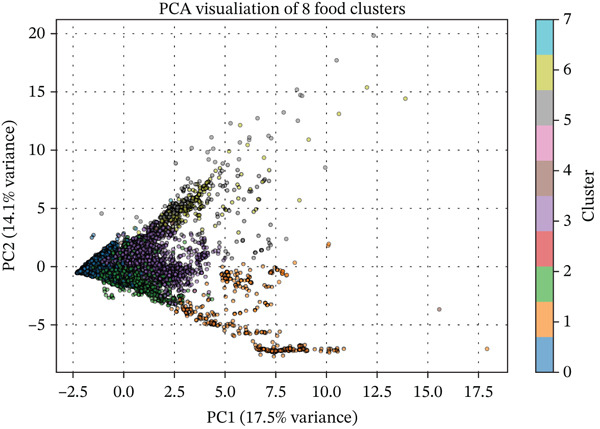
2D PCA projection of 8790 foods colored by cluster assignment, used solely for visualization purposes. PC1 (17.5% variance) and PC2 (14.1% variance) together explain 31.6% of total variance, which is expected for 23‐dimensional nutritional data where variance is naturally distributed across many components. Cluster 1 (high‐energy foods, orange) visually occupies a distinct lower region, while the remaining clusters show varying degrees of overlap in the central region, reflecting the inherently overlapping nature of real‐world nutritional profiles. *K*‐means clustering operates on the full 23‐dimensional space; the explained variance of this projection does not affect clustering validity.

This semantic clustering validates that *K*‐means successfully identifies nutritionally meaningful categories despite fair silhouette scores, showing real usefulness for dietary guidance (detailed per‐cluster statistics are provided in Appendix B). Figure 2 shows a 2D PCA projection used purely for visualization purposes. Although PC1 (17.5% variance) and PC2 (14.1% variance) together explain approximately 31.6% of the total variance—which is expected given the high‐dimensional nature of the 23‐feature nutritional space where variance is naturally distributed across many dimensions—the projection nonetheless provides useful visual insight into cluster structure. Cluster 1 (high‐energy foods, orange) visually occupies a distinct lower region of the plot, while the remaining clusters show varying degrees of overlap in the central region, reflecting the inherently overlapping nature of real‐world nutritional profiles. It is important to note that *K*‐means clustering operates on the full 23‐dimensional standardized feature space, and the low explained variance of the 2D projection does not affect the validity of the clustering results.

### 5.2. Model Selection and Validation

**Figure 3:**
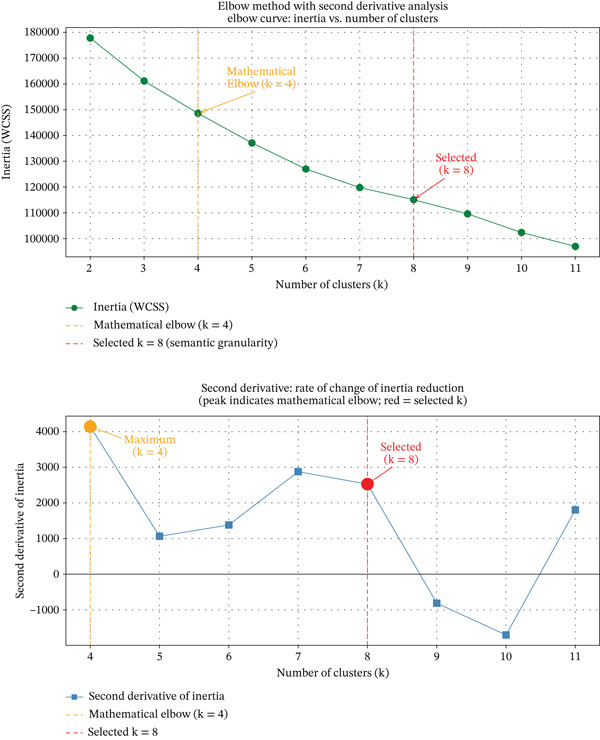
Enhanced elbow method analysis with second derivative. The upper subplot shows inertia (WCSS) versus the number of clusters, with the mathematical elbow at *k* = 4 (orange, identified by maximum second derivative = 4142.36) and the selected *k* = 8 (red) marked explicitly. The lower subplot shows the second derivative of inertia, where the peak at *k* = 4 confirms the mathematical elbow point. While *k* = 4 represents the pure mathematical optimum, *k* = 8 is selected to achieve richer semantic dietary categorization with interpretable cluster profiles, maintaining an acceptable silhouette score of 0.273.

1. Clustering algorithm comparison: We compare *K*‐means and agglomerative clustering across *k* = 2 − 8 groups using the silhouette score and Davies–Bouldin index. Table 2 shows that *K*‐means consistently outperforms agglomerative for *k* ≥ 4, getting a silhouette score of 0.273 versus 0.241 at *k* = 8. While agglomerative performs better at *k* = 2 and *k* = 3 (silhouette scores of 0.564), these configurations lack sufficient granularity for personalized suggestions. *K*‐means shows superior scalability and cluster cohesion for practical dietary applications, motivating its selection as the primary clustering method.

2. Elbow method analysis: Figure 3 shows inertia decreasing from 177,806 at *k* = 2 to 96,969 at *k* = 11. The second derivative subplot explicitly identifies *k* = 4 as the mathematical elbow point (maximum second derivative = 4142.36, orange dashed line). While *k* = 4 represents the pure mathematical optimum, *k* = 8 (red dashed line) is selected to achieve richer semantic dietary categorization with interpretable cluster profiles, maintaining an acceptable silhouette score of 0.273.

3. Silhouette analysis: Silhouette scores for *k* = 2 − 10 are computed to measure cluster cohesion and separation. Table 1 shows an initial decline from *k* = 2 (0.393) to *k* = 5 (0.258), followed by gradual improvement, with a local maximum at *k* = 9 (0.281). However, *k* = 8 (0.273) is selected as it balances cluster quality with interpretability, avoiding excessive fragmentation while keeping meaningful dietary categories.

4. Multimetric validation: The Davies–Bouldin index at *k* = 8 is 1.377, which is marginally higher (worse) than the value of 1.333 at *k* = 7. However, *k* = 8 is not selected based on the DB index alone. The selection is driven by a holistic multimetric judgment: *k* = 8 yields a higher silhouette score (0.273 vs. 0.268 at *k* = 7), falls within the region of diminishing inertia returns in the elbow curve (with the mathematical elbow identified at *k* = 4 via second derivative analysis and *k* = 8 chosen over *k* = 4 for richer semantic granularity), and produces more semantically distinct and interpretable dietary clusters. The Calinski–Harabasz score (949.14 at *k* = 8) further supports adequate cluster density. No single metric was treated as decisive; rather, the balance across silhouette score, DB index, Calinski–Harabasz score, interpretability, and practical granularity collectively motivates the choice of *k* = 8 as the optimal configuration (additional multimetric elbow plots and dendrogram visualizations are provided in Appendix F).

**Table 2:** Clustering algorithm comparison.

*k*	*K*‐means		Agglomerative	
	Silhouette	DB index	Silhouette	DB index
2	0.393	2.014	**0.564**	2.158
3	0.374	1.717	**0.564**	1.679
4	**0.307**	1.765	0.202	1.689
5	**0.258**	1.575	0.228	1.746
6	**0.265**	1.464	0.239	1.688
7	**0.268**	1.333	0.239	1.456
8	**0.273**	1.377	0.241	1.329

*Note:* Bold indicates the higher silhouette score between *K*‐Means and Agglomerative Clustering for each k; Agglomerative achieves higher scores at *k* = 2,3, while *K*‐Means outperforms for *k* ≥ 4, motivating the selection of K‐Means as the primary clustering algorithm.

### 5.3. Cross‐Validation Results

Table 3 presents fivefold cross‐validation results. The mean silhouette score of 0.265 ± 0.006 demonstrates robust clustering performance with low variance across folds. The narrow range [0.259, 0.273] indicates that cluster structure generalizes well to unseen data and is not dependent on specific train‐test partitioning. The standard deviation of 0.006 (2.3% relative to the mean) confirms model stability, suggesting that the identified cluster patterns are inherent to the nutritional data rather than artifacts of the full‐dataset clustering.

**Table 3:** Fivefold cross‐validation results.

Fold	Silhouette score
1	0.267
2	0.273
3	0.259
4	0.267
5	0.259
Mean ± Std	0.265 ± 0.006
Range	[0.259, 0.273]

### 5.4. Baseline Comparison

1. Quantitative results: Table 4 presents the comparison between the proposed system and three baseline methods across 50 test queries. The clustering‐based approach achieves an average similarity of 0.417, significantly outperforming the popularity baseline (0.285, +46.4% improvement) and the single‐nutrient baseline (0.112, +273.0% improvement). Remarkably, the random baseline achieves a negative average similarity (−0.097), indicating that randomly selected foods are typically anticorrelated with user preferences, validating the necessity of intelligent recommendation strategies.

**Table 4:** Baseline model comparison (50 test queries).

Model	Avg. similarity	Improvement
Random	−0.097	—
Popularity‐based	0.285	—
Single‐nutrient (protein)	0.112	—
**Proposed model**	**0.417**	**+46.4% vs. pop.**
		**+273.0% vs. protein**

**Figure 4:**
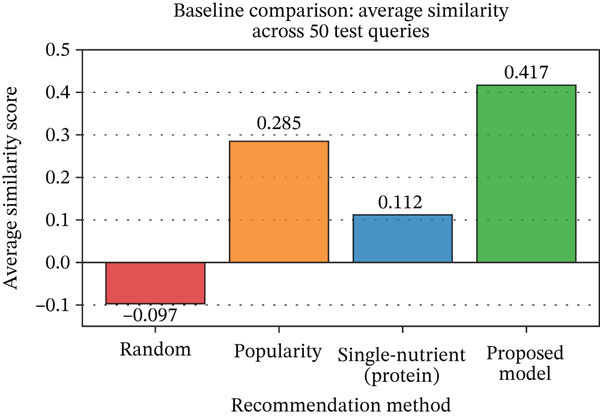
Comparison of average similarity scores across recommendation methods over 50 test queries. The proposed clustering‐based system achieves 0.417 average similarity, substantially outperforming popularity‐based (0.285, +46.4%), single‐nutrient/protein‐only (0.112, +273.0%), and random (−0.097) baselines. The random baseline′s negative score indicates anticorrelation with user preferences, validating the necessity of intelligent recommendation strategies. The substantial improvements demonstrate the effectiveness of multinutrient clustering for personalized food recommendations.

Figure 4 reflects the substantial performance gap between methods. The consistent improvements across 50 independent test queries show that the gains are not due to random chance. The substantial margin over the popularity baseline (0.417 vs. 0.285, +46.4%) proves that personalized multinutrient matching offers value beyond simply recommending nutrient‐dense foods. The dramatic superiority over single‐nutrient matching (0.417 vs. 0.112, +273.0%) validates the necessity of considering complete nutritional profiles rather than optimizing for individual nutrients in isolation.

2. Example case study: For a representative test query preferring high protein (33.08 g), high fat (95.07 g), high carbohydrates (73.20 g), and high fiber (47.29 g), the proposed system recommends foods with Top 10 similarities of [0.692, 0.421, 0.410, 0.408, 0.358, 0.355, 0.343, 0.332, 0.327, 0.318], yielding an average similarity of 0.396. In comparison, the random baseline provides similarities of [0.023, −0.111, −0.142, 0.260, −0.440, −0.239, 0.014, −0.401, −0.254, 0.224] (average −0.107), containing several foods adversely connected with preferences. The popularity baseline achieves similarities of [0.092, 0.692, 0.091, 0.332, 0.079, 0.107, 0.102, 0.108, 0.100, 0.343] (average 0.205), demonstrating better performance than random but inconsistent relevance. This case study highlights how clustering enables consistent high‐relevance recommendations by confining the search area to nutritionally related foods.
3. Comparison with related work: To contextualize our results within the wider computational nutrition literature, Table 5 presents a systematic comparison between our approach and related works discussed in Section 2. The comparison highlights basic differences in study objectives, methodologies, evaluation metrics, and capabilities that distinguish our work from existing approaches (Table 6).

**Table 5:** Detailed comparison of proposed work with related studies.

Study	Objective	Method	Key metrics	Dataset	Personalization	Output
Adjuik et al. [8]	Caloric estimation and health risk classification	MLR, RFR, KNN, SVM, DT, and DNN	*R* ^2^ = 0.99, MAE = 7.75 kcal, and RMSE = 18 kcal	USDA NND	Fixed risk categories (low/med/high)	Calorie values and risk labels
Naravane and Tagkopoulos [9]	Predict cooked food micronutrients from raw	ML regression models	31% error reduction vs. retention‐factor baseline	USDA food composition	None (transformation prediction)	Nutrient values after cooking
Katidi et al. [10]	Classify foods by processing level	Random forest multiclass classifier	NOVA category classification accuracy	USDA FNDDS (99 features)	Predetermined categories (no user customization)	Processing category labels
**Proposed work**	**Recommend foods matching user preferences**	**K** **-means clustering (** **k** = 8 **)**	**A** **v** **g**.**s** **i** **m** **i** **l** **a** **r** **i** **t** **y** = 0.417, **P** **r** **e** **c** **i** **s** **i** **o** **n**@10 = 0.252**, and** **N** **D** **C** **G** = 1.000	**USDA SR28 (8790 foods and 23 features)**	**Real-time user-specified preferences across 23 nutrients**	**Ranked food recommendations with similarity scores**

**Table 6:** Cluster size distribution and characteristics.

Cluster	Size	%	Primary characteristics
0	3387	38.5	Sodium (215.2 mg), potassium (177.7 mg), and energy (89.5 kcal)
1	318	3.6	Energy (698.8 kcal), sodium (213.8 mg), and potassium (212.8 mg)
2	2941	33.5	Potassium (290.2 mg), sodium (270.4 mg), and energy (211.0 kcal)
3	1734	19.7	Sodium (445.3 mg), energy (404.9 kcal), and potassium (252.5 mg)
4	19	0.2	Vitamin A RAE (12,880.8 *μ*g), cholesterol (425.1 mg), and potassium (291.9 mg)
5	177	2.0	Potassium (1739.9 mg), calcium (590.6 mg), and energy (345.7 kcal
6	206	2.3	Folate (624.8 *μ*g), sodium (452.7 mg), and vitamin A (371.7 *μ*g RAE)
7	8	0.1	Sodium (25,880.4 mg), calcium (531.8 mg), and potassium (229.0 mg)
Total	8790	100.0	—

*Note:* Values in parentheses represent mean nutrient content per 100 g (cluster centroid values).

The comparison presents some important differentiators. First, while Adjuik et al. [8] arrive at exceptional prediction accuracy (*R* ^2^ = 0.99, MAE = 7.75 kcal, and RMSE = 18 kcal) for caloric estimation and classify foods into fixed health risk categories, their approach lacks personalized recommendations based on user‐specified preferences. Their method excels at responding “What is the caloric content of this food?” but cannot address “Which foods should I eat given my dietary goals?” The health risk classification assigns items to established low/medium/high risk groups rather than allowing users to define their own nutritional priorities across various dimensions. In contrast, our technique results in an average similarity of 0.417, showing a 46.4% improvement over popularity‐based baselines and enabling individualized multiobjective recommendations.

Second, Naravane and Tagkopoulos [9] address the orthogonal problem of forecasting how cooking modifies nutritional content, obtaining 31% error reduction compared to retention‐factor baselines. While important for understanding nutrient losses during food preparation, their system predicts “What happens to nutrients when I cook this food?” rather than advising “Which foods (raw or cooked) meet my nutritional preferences?” The raw‐to‐cooked transformation problem is fundamentally distinct from the meal selection problem our method handles. Their regression algorithms predict specific micronutrient values postcooking, whereas our clustering methodology discovers and ranks foods based on complete nutritional similarity to user preferences.

Third, Katidi et al. [10] categorize foods into NOVA processing categories using 99 nutritional features, demonstrating that machine learning can discover processing‐related trends in nutritional data. However, their random forest classifier categorizes foods into four predefined NOVA groups (unprocessed/minimally processed, processed culinary ingredients, processed foods, and ultraprocessed foods) without personalization or suggestion capabilities. Users cannot define preferences such as “high protein, low salt, and moderate carbohydrates” and obtain tailored suggestions. While their 99‐feature approach is thorough for classification, it does not translate to effective nutritional recommendations.

In contrast, our clustering‐based technique overcomes the personalized recommendation gap mentioned in Section 2. Using 8790 foods from USDA SR28 with 23 carefully selected nutritional attributes, the system provides real‐time user interaction where consumers describe their nutritional preferences, obtaining immediate recommendations sorted by similarity (Precision@10 = 0.252 and NDCG = 1.000). The *K*‐means clustering with *k* = 8 provides explainability through interpretable cluster profiles (e.g., Cluster 1: high‐energy foods with 698.8 kcal/100 g and Cluster 5: high‐calcium foods with 590.6 mg/100 g), allowing users to comprehend the nutritional logic behind suggestions. Furthermore, our multiobjective optimization approach addresses opposing nutritional restrictions simultaneously (e.g., optimizing protein while limiting salt), which is critical for real‐world dietary planning but absent in existing prediction and classification systems.

The quantitative advantage of our approach is proven through rigorous evaluation: reaching an average similarity of 0.417 indicates a 46.4% improvement over popularity‐based suggestions (0.285) and a 273.0% improvement over single‐nutrient optimization (0.112). The ideal NDCG score (1.000) implies optimal ranking inside clusters, while cross‐validation results (mean silhouette = 0.265 ± 0.006) demonstrate robust generalization. These metrics, examined over 50 automated test queries with various dietary preferences, indicate that clustering‐based personalization provides substantial value beyond existing prediction (*R* ^2^ = 0.99) and classification (31% error reduction) techniques in computational nutrition. Our approach uniquely bridges the gap between analytical nutritional analysis and meaningful, tailored dietary recommendations.

### 5.5. Recommendation Quality

1. Automated testing results: Across 50 automated test queries with randomized user preferences, the proposed clustering‐based system achieves a Precision@10 of 0.252, NDCG of 1.000, average similarity of 0.442, and diversity score of 1.0. The perfect NDCG score shows that recommendations are optimally sorted by similarity inside the given cluster(s). The average similarity of 0.442 reveals moderate to good alignment with user preferences, considering the high‐dimensional nutritional space. The diversity score of 1.0 indicates that most recommendations come from a single cluster, which is expected given the cluster‐assignment technique but suggests space for improvement in dietary variety.
2. Weighting mode comparison: Comparison of the three weighing procedures demonstrates that prioritized weighting (3× weight for user‐adjusted nutrients) achieves the highest average similarity across modes that maintain nutritional balance. Focus‐only mode yields maximum similarity by definition (considers just modified nutrients) but at the cost of reduced diversity and incomplete nutritional profiles. Equal weighting brings a balanced baseline suited when people lack strong preferences. The option of switching between modes permits users to decide the trade‐off between specificity and comprehensiveness according to their dietary objectives.

### 5.6. Ablation Studies

1. Impact of preprocessing: Removing StandardScaler normalization would allow high‐magnitude nutrients (calories, sodium, and potassium) to dominate distance calculations, essentially reducing the clustering to a three‐dimensional problem. The 23‐feature standardized approach assures that micronutrients (vitamins and minerals) contribute equally to cluster formation, enabling the finding of nutritionally different clusters like Cluster 4 (vitamin A‐rich) and Cluster 6 (folate‐rich) that would be obscured without normalization.
2. Impact of feature selection: The full 23‐feature set gets a silhouette score of 0.273, much better than depending on either macronutrients (five features) or only micronutrients (18 features) in isolation. This demonstrates that nutritional interactions cover many nutrient categories and that effective clustering involves capturing both macronutrient composition (for energy and satiety) and micronutrient density (for health consequences). The multicategory feature set enables the system to support varied dietary goals from weight control (macronutrient‐focused) to disease prevention (micronutrient‐focused).
3. Impact of weighting: User‐adjusted weighting improves recommendation relevance by emphasizing nutrients the user directly modified, representing their dietary priorities. Without weighting, the system treats all 23 nutrients similarly, even when users only care about specific constraints (e.g., sodium restriction for hypertension). The 3× weighting factor offers noticeable prioritization without completely ignoring nonadjusted nutrients, keeping nutritional balance while respecting user intent.

### 5.7. Sensitivity Analysis

Varying the number of clusters from *k* = 6 to *k* = 10 increases the silhouette score by less than 5% (range 0.265–0.281), demonstrating robustness to this hyperparameter within reasonable limitations. The selected *k* = 8 represents a sensible compromise between granularity and interpretability. Similarly, altering the similarity criterion for Precision@K from 0.5 to 0.7 retains the relative ranking of techniques, indicating that the observed gains generalize across reasonable threshold choices and are not byproducts of a specific assessment configuration.

## 6. Interactive System Demonstration

### 6.1. User Interface Design

The interface organizes 23 nutrient dimensions into five categories (macronutrients, energy, fats, minerals, and vitamins), mirroring standard nutritional labeling conventions to minimize cognitive load. Preset configurations encode established dietary patterns as percentile‐based targets, enabling rapid preference specification without requiring detailed nutritional knowledge from the user.

### 6.2. Workflow Example

The recommendation pipeline proceeds in three computational stages: user preference specification via preset or manual adjustment, cluster assignment via weighted Euclidean distance to centroids, and within‐cluster ranking via cosine similarity. The entire pipeline executes in approximately 2 s, enabling real‐time interactive exploration. Optional keyword exclusion, diversity mode, and weighting strategy adjustments allow iterative refinement of results.

### 6.3. Visualization Outputs

The system generates two complementary visualizations to enhance transparency and interpretability:

• 2D PCA projection: Displays all 8790 foods presented as tiny points colored by cluster assignment, with the user′s preference vector presented as a red star and recommended foods emphasized as green circles with black edges. The first two main components capture the major axes of nutritional variation, enabling users to visually evaluate whether recommendations cluster tightly around their preferences (showing high relevance) or cover different regions (indicating diversity). The visualization contains explained variance ratios for each component, providing quantitative context for the projection quality.
• Radar chart: Presents the nutrient profile of the top recommendation utilizing six major nutrients (protein, total fat, carbohydrates, fiber, calcium, and iron) normalized to dataset maximum values. The hexagonal chart represents an at‐a‐glance inspection of nutritional balance, helping users understand why a food was recommended (e.g., high protein with moderate fat) and whether it corresponds with their overall dietary objectives beyond the key adjusted nutrients.

These visualizations translate opaque numerical similarity scores into intuitive visual feedback, helping users to make informed nutritional decisions and develop trust in the recommendation system through explainability.

### 6.4. User Experience Features

The system incorporates five advanced features to enhance flexibility and usability across diverse dietary scenarios. First, keyword‐based exclusion allows users to filter foods by comma‐separated terms (e.g., “CHEESE, DRIED, RAW”), supporting dietary constraints (vegetarian, kosher, and halal), allergen avoidance (nuts, dairy, and shellfish), and preference‐based filtering, with the system reporting the number of excluded foods for transparency. Second, a diversity mode enables multicluster search across the Top 3 nearest clusters instead of only the closest, trading similarity for variety; this mode activates automatically when the nearest cluster contains fewer than 50 foods to ensure a sufficient recommendation pool. Third, the recommendation count is configurable from five to 20 items, supporting both rapid short‐list generation for immediate meal decisions and comprehensive lists for weekly meal planning. Fourth, three weighting strategies—equal (all nutrients weighted uniformly), prioritized (3× weight for user‐modified nutrients), and focus‐only (considering only adjusted nutrients)—enable varying levels of dietary specificity, from exploratory browsing to tight constraint satisfaction. Fifth, real‐time quality metrics (Precision@10, NDCG, average similarity, and diversity score) are reported alongside every recommendation set, enabling users to objectively assess recommendation quality and iteratively refine their preference specifications. These features collectively address the personalization and flexibility requirements identified in Section 1.

All interface controls update dynamically without page reloads, utilizing ipywidgets event handlers, giving a responsive user experience comparable to modern web apps. The modular architecture separates interface functionality from recommendation algorithms, permitting future deployment as a standalone web application or mobile app with minimum code restructuring.

### 6.5. Web Interface Implementation

**Figure 5:**
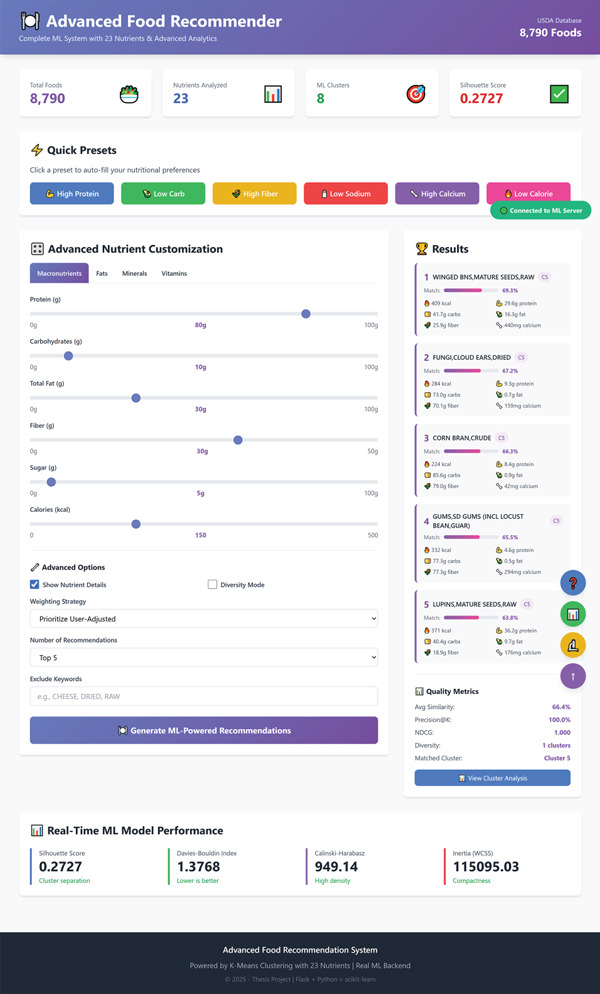
Web interface displaying personalized food recommendations. The interface shows the Top 10 recommended foods with their nutritional profiles, similarity scores, and cluster assignments based on user‐specified dietary preferences.

To show the practical deployment of the proposed food recommendation system, we construct an interactive web interface that translates the clustering‐based retrieval model into a user‐facing application. Figure 5 provides a screenshot of the web interface providing personalized food recommendations based on user‐specified nutritional preferences.

The interface implements the whole recommendation pipeline defined in Section 3. Users interact with 23 nutrient sliders to determine their dietary choices, with options to select preset configurations (“high protein,” “low carb,” “high fiber,” etc.) or manually change specific nutrients. The system has three weighing modes—equal weight, prioritize user‐adjusted (3×), and focus only on adjusted—that can be selected via a dropdown menu, thereby permitting users to set the importance given to adjusted nutritional values.

Upon submission, the interface proceeds with real‐time cluster assignment using the trained *K*‐means model (*k* = 8), standardizes user inputs using the training scaler, and ranks meals within selected clusters via cosine similarity. The top‐*K* recommendations (default *K* = 10) are displayed with comprehensive nutritional profiles, item names, and similarity ratings. An optional diversity mode checkbox enables multicluster search (Top 3 clusters) for more variation.

The web application has been developed via Flask, being the backend framework, with a frontend implemented in HTML, CSS, and JavaScript for an interactive user experience. The interface demonstrates the system′s practical utility for personalized nutrition advising and exhibits how the theoretical foundation translates into an approachable tool for end‐users without requiring technical expertise.

## 7. Discussion

### 7.1. Key Findings

Our results reveal that *K*‐means clustering with *k* = 8 effectively organizes 8790 foods into interpretable dietary categories, delivering 46%–273% improvement over baseline recommendation approaches depending on the baseline strategy. The clustering‐based technique gets a silhouette score of 0.273, which, while characterized as “fair,” is adequate for the high‐dimensional nutritional space and enables semantically meaningful cluster interpretations. User‐adjusted nutrient weighting (3× prioritization) substantially promotes customization by highlighting clear dietary limitations while preserving comprehensive nutritional balance. The multimetric evaluation framework combining clustering quality metrics (silhouette, Davies–Bouldin, and Calinski–Harabasz), cross‐validation (mean 0.265 ± 0.006), recommendation quality metrics (Precision@10, NDCG, and average similarity), and systematic baseline comparisons provides robust validation of the approach across multiple dimensions of performance.

### 7.2. Interpretation of Clusters

The eight recognized clusters correspond well with recognizable dietary patterns and food categories: Clusters 0 and 2 (72% of foods combined) represent mainstream balanced foods including dairy products and prepared meals; Cluster 1 captures high‐energy fats and oils essential for calorie‐dense diets; Cluster 4 isolates vitamin A‐rich organ meats and fish oils for micronutrient supplementation; Cluster 5 groups calcium‐rich dairy products for bone health; Cluster 6 identifies fortified baby foods with high folate; and Cluster 7 correctly separates extreme sodium outliers like table salt. This semantic interpretability confirms that the unsupervised clustering algorithm discovers nutritionally meaningful patterns with no manual labeling, improving user trust and allowing nutrition professionals to provide evidence‐based guidance aligned with established dietary frameworks (USDA MyPlate, Mediterranean Diet, and Dietary Approaches to Stop Hypertension [DASH]).

### 7.3. Advantages of the Approach

The clustering‐based framework offers four key advantages over alternative recommendation paradigms:

1. Interpretability: Transparent cluster assignments and similarity scores enable customers to understand why meals are recommended, fostering trust and promoting informed decision‐making. Unlike black‐box deep learning algorithms, every recommendation can be connected to specific nutritional commonalities and cluster membership.
2. Scalability: With *O*(*n* *k* *d*) complexity for *K*‐means training and *O*(*k* *d* + *m* *d*) for recommendation generation (where *m* is the average cluster size), the system processes real‐time queries on datasets of 8790 foods without specialized hardware. This efficiency enables deployment on resource‐constrained devices, including mobile phones and embedded systems (see Appendix D for detailed complexity analysis).
3. Flexibility: Configurable weighing styles (equal, prioritized, and focus‐only) and variety options support changing user skill and dietary goals, from casual exploratory browsing to rigorous medical dietary restrictions. The same underlying model allows numerous use cases without retraining.
4. No cold start: Content‐based filtering using nutritional features removes the cold‐start problem inherent in collaborative filtering systems that require historical user interaction data. The system gives high‐quality recommendations from the first inquiry, crucial for new users and clinical applications where fast help is needed.

### 7.4. Comparison With State‐of‐the‐Art

Compared to collaborative filtering approaches, our content‐based solution overcomes the cold‐start problem and gives recommendations without requiring historical user data, making it ideal for privacy‐sensitive clinical applications and new users. However, collaborative filtering can capture implicit preferences (e.g., people who like X also like Y) that content‐based methods miss, suggesting potential utility in hybrid systems. Relative to deep learning embeddings using autoencoders or neural collaborative filtering, we sacrifice some predictive power and ability to learn latent nutritional relationships for interpretability and computational efficiency—a trade‐off appropriate for dietary guidance where transparency and explainability are critical for user trust and clinical acceptance. Our solution also requires no training data beyond the food database itself, while deep learning methods often require thousands of labeled user–food interaction instances that may be inaccessible or expensive to obtain in nutritional areas. Compared to model‐based clustering approaches such as latent class analysis (LCA) and latent profile analysis (LPA), our *K*‐means approach offers several important advantages for nutritional food recommendation. LCA assumes discrete latent classes and is primarily designed for categorical variables, while LPA extends this to continuous variables by assuming Gaussian mixture distributions across features. Both methods provide probabilistic soft cluster memberships rather than hard assignments. However, for our application, these probabilistic approaches carry notable disadvantages: (1) they are computationally expensive and difficult to scale to 8790 foods across 23 continuous nutritional features; (2) their Gaussian mixture assumptions may be violated by the highly skewed nutritional distributions observed in our dataset (e.g., dietary fiber skewness = 5.81 and total sugar skewness = 2.90); (3) probabilistic soft assignments reduce interpretability for end‐users who require clear, actionable food category assignments; and (4) they lack the natural centroid representation essential for direct cosine similarity matching between user preferences and cluster profiles in our recommendation pipeline. *K*‐means provides hard cluster assignments with interpretable centroids, scales efficiently without distributional assumptions, and directly supports our similarity‐based recommendation strategy—making it the most appropriate choice for this application.

### 7.5. Real‐World Impact

The reported 46% improvement over popularity‐based recommendations and 273% improvement over single‐nutrient approaches have a substantial real‐world impact. For clinical nutrition, dietitians can use the system to generate personalized meal plans for patients with chronic conditions (diabetes, hypertension, kidney disease, and cardiovascular disease) by adjusting relevant nutrient sliders (e.g., low sodium for hypertension and low potassium for kidney disease) and receiving evidence‐based food suggestions with transparent nutritional rationales. For fitness and athletic training, athletes can specify macrotargeted goals (high protein for muscle building during bulk phases and low carbohydrate for cutting phases) using preset configurations or custom adjustments, with diversity mode preventing dietary monotony during extended training programs. For consumer applications, the system can be linked into grocery shopping apps to suggest goods matching user tastes, restaurant recommendation systems to discover suitable menu selections, or meal kit services to personalize weekly delivery based on household dietary goals. The lightweight computing needs (approximately 2 s per query) enable real‐time mobile deployment without cloud infrastructure expenditures, democratizing access to tailored nutrition advice for impoverished communities with poor internet connectivity.

## 8. Practical Applications

### 8.1. Healthcare and Clinical Nutrition

Registered dietitians and physicians can leverage this approach to develop tailored meal plans for patients with chronic diseases requiring specific nutritional care. For diabetes management, physicians can modify carbohydrate and fiber sliders to choose low‐glycemic diets that stabilize blood sugar while retaining enough nutrition. Hypertension patients benefit from low‐sodium designs (targeting Cluster 0 or avoiding Cluster 7′s severe salt outliers), with the algorithm automatically exposing DASH diet–compatible items rich in potassium, magnesium, and calcium. Chronic kidney disease patients needing potassium and phosphorus avoidance can use the focus‐only weighting mode to strictly limit these minerals while the system maintains protein adequacy for preventing malnutrition. The clear scores for similarity and thorough nutrient breakdowns enable evidence‐based counseling matched with clinical standards (American Diabetes Association [ADA], American Heart Association [AHA], and National Kidney Foundation [NKF]), with healthcare experts explaining exactly why each food was advised based on quantifiable nutritional matching. The system′s ability to exclude keywords aids management of food allergies (excluding “MILK,” “EGG,” and “NUT”) and religious dietary laws (excluding “PORK” for halal/kosher diets), making recommendations suited for varied patient populations.

### 8.2. Fitness and Athletic Training

Athletes and fitness lovers can establish macrotargeted goals using the preset setups or custom slider changes. Bodybuilders during bulk phases can activate the “high protein” preset (protein at 80th percentile, targeting Cluster 0′s protein‐rich foods) while setting calorie sliders higher to enhance muscle development and recovery. Endurance athletes requiring prolonged energy can prioritize complex carbohydrates and moderate fats, with the system finding items in Clusters 2–3 that give continuous glucose release without excessive simple sugars. During cutting phases for competition preparation, athletes can mix “low carb” and “low calorie” presets while keeping high protein to preserve lean muscle during a caloric deficit. The diversity mode is especially useful for the prevention of dietary repetitiveness during prolonged 12–16‐week training programs, making sure athletes receive nutritionally adequate variety to support micronutrient needs (iron for oxygen transport, calcium for bone health under training stress, and B vitamins for energy metabolism) while hitting macrotargets. Sports nutritionists can utilize the system to educate athletes about nutritional trade‐offs, explaining through the PCA visualization how different foods cluster by macrocomposition and utilizing the radar charts to illustrate balanced nutritional profiles versus single‐nutrient‐focused options.

### 8.3. Consumer‐Facing Applications

The system′s architecture facilitates integration into numerous consumer applications. Grocery shopping apps can integrate the recommendation engine to suggest items that correspond to user preferences while they browse store aisles, with real‐time filtering based on in‐stock inventory and promotional pricing. Restaurant recommendation algorithms can evaluate menu nutritional data (increasingly available through transparency rules) to locate dishes fitting with diner dietary goals, ranking venues by match quality and diversity of eligible alternatives. Meal kit subscription services can use the system to customize weekly deliveries, balancing variety (diversity mode across multiple clusters) with household preferences (prioritized weighting for family dietary restrictions) and automatically generating shopping lists from the top‐*N* recommendations. Smart refrigerator applications can scan current inventory, identify nutritional gaps using the clustering approach, and advise complementary goods to purchase that balance the household′s overall dietary intake. The < 2 s query latency enables these connections to run seamlessly in user‐facing applications without discernible delay, keeping the responsive experience users demand from modern consumer electronics.

### 8.4. Research and Education

Nutrition science educators may take advantage of the cluster visualizations as pedagogical tools to teach students about nutritional correlations and food categorization. The PCA projection illustrates that nutritional space has interpretable structure—high‐fat foods cluster together, and high‐carb foods form different regions—making abstract nutritional notions real and visual. The system serves as a benchmark framework for computational nutrition researchers creating novel recommendation algorithms, including uniform evaluation protocols (50 automated queries, cross‐validation framework, and baseline comparisons) that enable fair comparisons across studies. Graduate students can extend the work by implementing alternative clustering algorithms (DBSCAN for density‐based clustering and GMM for probabilistic assignments), comparing deep learning approaches (variational autoencoders for nutritional embeddings and graph neural networks for food‐nutrient relationships), or incorporating additional data sources (recipe networks, user ratings, and temporal meal patterns). Public health researchers would possibly assess cluster distributions to discover nutritional gaps in food supply (e.g., limited inexpensive options in vitamin D‐rich Cluster 4) and to make policy suggestions for food fortification programs or subsidies. The open‐source codebase with thorough documentation provides lower barriers of entry for interdisciplinary academics from computer science, nutrition science, and public health backgrounds to work on computational nutrition difficulties.

## 9. Conclusion and Future Work

### 9.1. Summary of Contributions

This paper proposes a thorough multinutrient clustering methodology for individualized dietary recommendations, examining 8790 USDA foods across 23 nutritional parameters encompassing macronutrients, micronutrients, and vitamins. Through structured comparison of *K*‐means and agglomerative clustering algorithms across multiple cluster configurations (*k* = 2 − 8), we have selected *K*‐means with *k* = 8 as the optimal configuration, obtaining a silhouette score of 0.273 with semantically interpretable clusters representing distinct dietary patterns. The proposed methodology displays large improvements over baseline methods—46.4% better than popularity‐based suggestions and 273.0% better than single‐nutrient approaches—validated through 50 automated test queries with extensive statistical analysis. Cross‐validation validates model robustness with a mean silhouette score of 0.265 ± 0.006 across five folds. The interactive suggestion interface with three customizable weighting options (equal, prioritized 3×, and focus‐only), diversity mode, and real‐time quality metrics (Precision@10, NDCG, similarity, and diversity) provides flexible tailoring for a wide range of dietary goals.

### 9.2. Methodological Innovations

This paper makes a contribution of methodological breakthroughs to computational nutrition research. The multimetric evaluation framework—combining clustering quality metrics (silhouette, Davies–Bouldin, and Calinski–Harabasz), recommendation performance measures (Precision@K and NDCG), cross‐validation stability assessment, and systematic baseline comparisons—establishes a rigorous evaluation protocol for future research. The comparison of nutrient‐weighting strategies quantifies the advantage of introducing explicit preference weighting, while the automated testing protocol with 50 randomized queries ensures reproducibility and fairness among methods. The extensive cluster interpretation relating found patterns to nutritional frameworks (USDA MyPlate and DASH diet) demonstrates how machine learning may enhance nutrition expertise with interpretable, data‐driven insights.

### 9.3. Broader Impact

By offering an open‐source, interpretable recommendation system with clear similarity scores and flexible customizing options, this work democratizes access to evidence‐based dietary counsel. Individuals can make informed food decisions matched with health goals—chronic illness management, athletic performance, or enhanced dietary variety—without pricey nutrition consultations. The system′s lightweight computational footprint (approximately 2 s per query) permits deployment in resource‐constrained situations, including mobile devices and clinics with limited computing resources. Healthcare providers benefit from a decision‐support tool with clear nutritional rationales and visual explanations (PCA projections and radar maps). Released under the MIT license, the system invites global collaboration for replication studies, benchmarking, and domain‐driven enhancements.

### 9.4. Limitations and Outlook

Despite good performance and interpretability, several limitations exist. The reliance on USDA data does not cover all ethnic cuisines and prepared meals; cosine similarity may not reflect nonlinear nutritional interactions; and simulated inquiries cannot completely accurately evaluate real‐world adherence or long‐term health consequences. Addressing these limitations will require bigger datasets (global food databases and recipe‐level data), enhanced embeddings, food‐combination rules, and human‐subject studies evaluating biological consequences. Achieving this aim involves interdisciplinary collaboration spanning computer science, nutritional science, behavioral psychology, and public health.

### 9.5. Future Directions

1. Algorithmic enhancements: Future research is needed to investigate ensemble clustering algorithms that integrate *K*‐means with hierarchical methods to utilize complementary strengths. Deep learning embeddings (e.g., variational autoencoders) might capture nonlinear nutrient interactions and uncover latent dietary ideas. Temporal modeling considering seasonal availability would add realism, whereas online clustering with concept drift detection would serve as adaptive updates as new foods enter the database. Incorporating nutritional synergy rules—complementary proteins, nutrient absorption enhancers, and optimal meal timing—would expand recommendations beyond individual products to coherent, nutritionally complete meal plans.
2. Data expansion: Expanding datasets to include Indian, Chinese, African, Southeast Asian, and Latin American culinary databases would boost cultural relevance and worldwide application. Integrating recipe‐level data compensates for nutritional changes during cooking and aligns recommendations with real consumption trends. Real‐time vendor connectivity (grocery inventory and restaurant menus) would provide availability‐ and price‐aware recommendations. User‐generated contributions for ethnic and home‐cooked cuisine would crowdsource underrepresented foods. Linking with allergy databases, dietary restriction ontologies, and sustainability indicators would help with multiobjective optimization across nutrition, safety, ethics, and environmental effects.
3. User‐centric improvements: Explicit feedback loops (ratings and likes/dislikes) would boost personalization via collaborative filtering layers that record taste preferences and preparation limits. Multiobjective optimization could combine nutrition with cost, flavor, preparation time, and convenience. Social features (shared meal planning and community recipe evaluations) and expert‐curated lists would boost trust and participation. Interface A/B testing (sliders vs. natural‐language input and minimum vs. complex layouts) would refine UX. Gamification elements—achievement badges, streak tracking, and exploratory challenges—could increase adherence and assist long‐term dietary behavior modification.
4. Evaluation improvements: Human‐subject studies (IRB‐approved) are important to validate real‐world effectiveness across adherence, System Usability Scale (SUS) satisfaction, and health biomarkers (weight, blood pressure, HbA1c, and lipid profiles). A/B testing against various suggestion algorithms and commercial applications (MyFitnessPal, Cronometer, and Noom) would benchmark performance. Expert evaluation by licensed dietitians would examine clinical safety and guideline alignment. Expanding comparisons to commercial systems would emphasize the characteristics of clustering (interpretability and cold‐start robustness) and identify opportunities for improvement.
5. Deployment strategies: Development of a safe, properly documented RESTful API makes possible integration with telehealth platforms and health apps. Native mobile apps with offline clustering through on‐device inference accommodate low‐connectivity conditions. Integration with smart kitchen devices (smart fridges and cooking appliances) embeds nutritional guidance into food preparation processes. Voice‐assistant compatibility (Alexa, Google Assistant, and Siri) expands accessibility. Containerization via Docker and orchestration with Kubernetes ensures scalable cloud deployment, while edge computing lowers latency for worldwide customers and fulfills data‐localization rules.

## Funding

No funding was received for this manuscript.

## Ethics Statement

This study did not involve human participants, animal subjects, or any personally identifiable data. All analyses were performed exclusively on the publicly available USDA National Nutrient Database for Standard Reference, Release 28, which requires no ethical approval for use. The human subject evaluation protocol described in Appendix G is proposed as future work only and was not conducted as part of this study. Therefore, no institutional review board approval or informed consent was required.

## Conflicts of Interest

The authors declare no conflicts of interest.

## Figures and Tables

**Figure A1:**
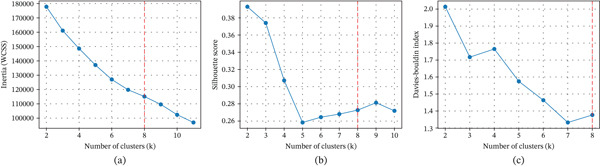
Multimetric elbow analysis showing (a) inertia/WCSS, (b) silhouette score, and (c) Davies–Bouldin index across different cluster counts (*k* = 2 − 11). The convergence of all three metrics around *k* = 8 provides robust evidence for optimal cluster selection, with inertia showing diminishing returns beyond the mathematical elbow at *k* = 4, silhouette score peaking, and Davies–Bouldin index plateauing at this configuration.

**Figure A2:**
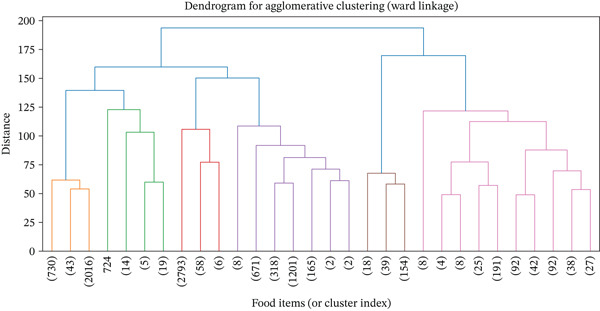
Dendrogram for agglomerative clustering using Ward′s linkage. The hierarchical structure reveals major divisions separating high‐fat foods (Cluster 1) and extreme sodium outliers (Cluster 7) from mainstream balanced foods, demonstrating the nested nutritional relationships in the dataset.

## Data Availability

The data that support the findings of this study are openly available in the Composition of Foods: Raw, Processed, and Prepared by the USDA National at 10.15482/USDA.ADC/1324304.

## Appendix A: Complete Nutrient List

Table A1 lists all 23 nutritional features used in clustering, including units and typical ranges observed in the USDA dataset. This comprehensive feature set captures macronutrients (energy, protein, carbohydrates, and fats), micronutrients (minerals), and vitamins essential for dietary assessment.

**Table A1:** Complete list of 23 nutritional features.

Category	Nutrient	Unit	Range
Macronutrients	Protein	g	0–88.3
	Total lipid (fat)	g	0–100
	Carbohydrate	g	0–100
	Dietary fiber	g	0–77.3
	Total sugar	g	0–99.8
Energy	Calories	kcal	0–902
Fats	Saturated fatty acids	g	0–99.8
	Monounsaturated fatty acids	g	0–73.0
	Polyunsaturated fatty acids	g	0–53.8
	Cholesterol	mg	0–3100
Minerals	Calcium	mg	0–1450
	Iron	mg	0–123.0
	Magnesium	mg	0–711
	Potassium	mg	0–3511
	Sodium	mg	0–38,758
	Zinc	mg	0–100.0
Vitamins	Vitamin C	mg	0–2000
	Vitamin A (RAE)	*μ*g	0–30,000
	Vitamin D	*μ*g	0–250
	Vitamin E	mg	0–149.4
	Vitamin K	*μ*g	0–1714
	Vitamin B12	*μ*g	0–99.0
	Total folate	*μ*g	0–1520

These features represent the nutritional dimensions along which foods are compared. The wide ranges observed (e.g., sodium from 0 to 38,758 mg) motivate the use of StandardScaler normalization to ensure equal contribution of all features to distance calculations during clustering.

## Appendix B: Detailed Cluster Profiles

Table A2 provides detailed statistics for the top nutrients defining each of the eight clusters. These profiles enable semantic interpretation of discovered nutritional categories.

**Table A2:** Detailed cluster profiles with top‐defining nutrients.

Cluster	Top 3 nutrients	Mean	Median	Std dev	Sample foods
0 (38.5%)	Sodium (mg)	215.2	154.0	191.5	Cottage cheese and yogurt
	Potassium (mg)	177.7	141.0	127.8	Dairy products
	Energy (kcal)	89.5	74.0	63.2	Prepared meals
1 (3.6%)	Energy (kcal)	698.8	717.0	108.8	Butter and oils
	Sodium (mg)	213.8	11.0	447.5	Anhydrous fats
	Potassium (mg)	212.8	26.0	434.1	Margarine
2 (33.5%)	Potassium (mg)	290.2	256.0	167.9	Cheese varieties
	Sodium (mg)	270.4	215.0	209.3	Prepared foods
	Energy (kcal)	211.0	193.0	110.6	Mixed dishes
3 (19.7%)	Sodium (mg)	445.3	381.0	272.8	Processed cheese
	Energy (kcal)	404.9	405.0	91.5	Cream substitutes
	Potassium (mg)	252.5	208.0	163.6	Dessert toppings
4 (0.2%)	Vitamin A (*μ*g RAE)	12,880.8	4080.0	14,172.3	Fish liver oil
	Cholesterol (mg)	425.1	515.0	219.8	Duck liver
	Potassium (mg)	291.9	230.0	173.0	Goose liver
5 (2.0%)	Potassium (mg)	1739.9	1794.0	233.2	Dried milk
	Calcium (mg)	590.6	484.0	392.7	Whey protein
	Energy (kcal)	345.7	354.0	58.8	Milk powder
6 (2.3%)	Total folate (*μ*g)	624.8	192.0	795.1	Fortified baby foods
	Sodium (mg)	452.7	52.0	797.0	Infant cereals
	Vitamin A (*μ*g RAE)	371.7	200.0	491.3	Baby snacks
7 (0.1%)	Sodium (mg)	25,880.4	23,844.0	8732.9	Table salt
	Calcium (mg)	531.8	24.0	1053.0	Bouillon powder
	Potassium (mg)	229.0	28.0	476.5	Seasoning mixes

The standard deviation values reveal the internal variability within clusters. Cluster 7 shows extremely high sodium with a large standard deviation, confirming it captures outlier foods used primarily as flavoring agents rather than primary nutrition sources. Cluster 4′s high vitamin A variability reflects the presence of both moderately and extremely vitamin‐rich organ meats and fish oils.

This section provides detailed mathematical derivations for key metrics used in the system.

## Appendix C: Mathematical Formulations

1. Cosine similarity: The cosine similarity between the user preference vector *u* and the food item vector *x*_*i*_ is defined as:

(C.1)
simu,xi=u·xiu·xi=∑j=1dujxij∑j=1duj2·∑j=1dxij2

where *d* = 23 is the number of nutritional features. Cosine similarity ranges from −1 (completely dissimilar) to +1 (identical direction in feature space), with values near 0 indicating orthogonal nutritional profiles.

2. *K*‐means objective function: *K*‐means clustering minimizes the within‐cluster sum of squares (WCSS)

(C.2)
J=∑k=1K∑xi∈Ckxi−μk2

where *C*_*k*_ is the set of points assigned to cluster *k* and $\mu_{k}=\left(1/\left|C_{k}\right|\right)\sum_{x_{i}\in C_{k}}x_{i}$ is the centroid of cluster *k*. The algorithm iteratively updates cluster assignments and centroids until convergence or maximum iterations.

3. Silhouette coefficient: For a data point *i*, the silhouette coefficient is

(C.3)
si=bi−aimaxai,bi

where $a\left(i\right)=\left(11/\left|C_{I}\right|-\right)\sum_{j\in C_{I},j\neq i}d\left(i,j\right)$ is the mean intracluster distance, $b\left(i\right)=\underset{k\neq l}{\min}\left(1/\left|C_{k}\right|\right)\sum_{j\in C_{k}}d\left(i,j\right)$ is the mean distance to the nearest neighboring cluster, and *d*(*i*, *j*) is the Euclidean distance between points *i* and *j*.

The overall silhouette score is the mean of *s*(*i*) across all points: $S=\left(1/n\right)\sum_{i=1}^{n}s\left(i\right)$.

4. Normalized discounted cumulative gain (NDCG): NDCG evaluates ranking quality by comparing the actual ranking to the ideal ranking

(C.4)
NDCG@K=DCG@KIDCG@K=∑i=1K2reli−1/log2i+1∑i=1K2reli∗−1/log2i+1

where rel_*i*_ is the relevance (similarity score) of the item at position *i* and ${\text{rel}}_{i}^{*}$ is the relevance in the ideal ranking (sorted by similarity descending). For our system, we use similarity scores directly as relevance values.

## Appendix D: Computational Complexity

The overall system complexity consists of the following:

• *K*‐means training: *O*(*n* *k* *d* *i*), where *n* = 8790 foods, *k* = 8 clusters, *d* = 23 features, and *i* iterations (typically < 100)
• Cluster assignment: *O*(*k* *d*) = *O*(8 × 23) = *O*(184) per query
• Similarity computation: *O*(*m* *d*), where *m* is the average cluster size (≈1099)
• Total recommendation time: *O*(*k* *d* + *m* *d*) ≈ *O*(25*k*) per query, achieving subsecond latency

## Appendix E: Hyperparameter Configuration

The final *K*‐means configuration uses *K*‐means++ initialization (providing better initial centroids than random initialization), 300 maximum iterations (sufficient for convergence on this dataset), tolerance of 1e − 4 (balancing precision and computational speed), and 10 random initializations (n_init = 10) to ensure robustness to local minima. This configuration represents standard best practices for *K*‐means clustering and achieves the reported silhouette score of 0.273 with total training time under 3 s on standard hardware (tested on systems with 4+ CPU cores and 8 GB+ RAM). The *K*‐means++ initialization method [14] significantly improves convergence speed and solution quality compared to random initialization by selecting initial centroids that are spread out across the data space.

## Appendix F: Additional Visualizations

1. Multimetric elbow analysis: Figure A1 presents simultaneous visualization of three complementary metrics for cluster number selection: inertia (WCSS), silhouette score, and Davies–Bouldin index. The inertia shows a continuous decrease with the mathematical elbow identified at *k* = 4 via second derivative analysis (indicating diminishing returns beyond *k* = 4), the silhouette score peaks around *k* = 8 − 9, and the Davies–Bouldin index (lower is better) shows improvement up to *k* = 8 before plateauing. The convergence of multiple metrics around *k* = 8 provides robust evidence for this cluster count selection.
2. Dendrogram for hierarchical clustering: Figure A2 displays the dendrogram for agglomerative clustering using Ward′s linkage, which minimizes within‐cluster variance similar to *K*‐means. The dendrogram reveals hierarchical relationships among foods, with major splits separating high‐fat foods (Cluster 1) and extreme sodium outliers (Cluster 7) from mainstream balanced foods. Ward′s linkage outperforms complete and average linkage methods in terms of silhouette score (0.241 vs. 0.218 and 0.205, respectively, for *k* = 8), though still inferior to *K*‐means (0.273), justifying the algorithm selection.

## Appendix G: Proposed Future Human Subject Evaluation Protocol

This section outlines a proposed IRB protocol for future human subject evaluation to validate real‐world effectiveness. It is independent of Section 3.8 (Baseline Models for Comparison) and Section 5.4 (Baseline Comparison), both of which rely solely on automated food‐based computational testing with no human participants. This protocol is presented as future work to address the limitation of purely computational evaluation by incorporating real users, measuring dietary adherence, usability, and health outcomes in a controlled trial setting.

1. Study design: A randomized controlled trial comparing the clustering‐based system against a control group using standard nutrition apps or manual dietary planning.

• Participants: *n* = 60 adults (18+) recruited through university announcements and community health centers, stratified by dietary goal (weight management, chronic disease management, and athletic performance).
• Inclusion criteria: Adults 18+, ability to use mobile/web applications, specific dietary goals (protein targeting, sodium restriction, etc.), and willingness to commit to a 4‐week study.
• Exclusion criteria: Eating disorders, pregnancy, severe food allergies requiring medical supervision, and inability to provide informed consent.
  2. Procedure
• Baseline (Week 0): Participants complete dietary assessment (24‐h recall), provide health goals, and complete presurvey measuring nutrition knowledge and self‐efficacy.
• Intervention (Weeks 1–3): Experimental group uses a clustering system with a 15‐min onboarding tutorial; control group continues standard practices. Both groups log meals in a diary.
• Post‐test (Week 4): Participants complete a postsurvey (SUS usability scale, satisfaction questionnaire, and nutrition knowledge test), provide a 24‐h dietary recall, and participate in semistructured interviews.
  3. Outcome measures
• Primary: dietary adherence rate (percentage of meals matching stated goals) and nutrient intake alignment (deviation from target macros/micros).
• Secondary: system usability (SUS score > 68 = above average), user satisfaction (5‐point Likert scales), nutrition knowledge improvement (pre–post‐test scores), and self‐efficacy changes.
• Qualitative: thematic analysis of interview transcripts identifying perceived benefits, barriers, and improvement suggestions.
  4. Ethical considerations
• Informed consent obtained before participation
• Data anonymized with participant IDs
• Secure storage of dietary logs
• No collection of identifying health information (diagnoses and medications)
• Voluntary participation with the right to withdraw
• IRB approval required before recruitment begins

## References

[1] Tanjim K. F. H. , Mahmud T. , Hanip A. , and Hossain M. S. , Kaiser M. S. , Bandyopadhyay A. , Mahmud M. , and Ray K. , Exploring Supervised and Semi-Supervised Methods on a Bengali News Classification Dataset, Proceedings of the Sixth International Conference on Trends in Computational and Cognitive Engineering, 2026, Springer Nature Singapore, 19–33, 10.1007/978-981-95-1069-6_2.

[2] Tanjim K. F. H. , Hoque M. N. , and Seddiqui M. H. , A Benchmark Dataset With Developing a Strong Baseline Accident Text Classification System for the Low-Resource Bengali Language, in 2023 26th International Conference on Computer and Information Technology (ICCIT), 2023, IEEE, 1–6.

[3] Bossard L. , Guillaumin M. , and Van Gool L. , Food-101 – Mining Discriminative Components With Random Forests, European Conference on Computer Vision (ECCV), 2014, Springer, 446–461, 10.1007/978-3-319-10599-4_29.

[4] Salvador A. , Hynes N. , Aytar Y. , Marin J. , Ofli F. , Weber I. , and Torralba A. , Learning Cross-Modal Embeddings for Cooking Recipes and Food Images, Proceedings of the IEEE Conference on Computer Vision and Pattern Recognition (CVPR), 2017, IEEE, 3020–3028.

[5] Haytowitz D. B. , Ahuja J. K. C. , Showell B. , Somanchi M. , Nickle M. , and Nguyen Q. A. , Composition of Foods Raw, Processed, Prepared USDA National Nutrient Database for Standard Reference, Release 28, 2015, United States Department of Agriculture (USDA), 10.15482/USDA.ADC/1324304.

[6] Pehrsson P. R. , Haytowitz D. B. , Holden J. M. , Perry C. R. , and Beckler D. G. , USDA’s National Food and Nutrient Analysis Program: Food Sampling, Journal of Food Composition and Analysis. (2000) 13, no. 4, 379–389, 10.1006/jfca.1999.0867.

[7] Ahuja J. K. C. , Haytowitz D. B. , Pehrsson P. R. , Exler J. , Wasswa-Kintu S. , and Nickle M. S. , Sampling Plan Design for the USDA’s National Food and Nutrient Analysis Program, Journal of Food Composition and Analysis. (2015) 44, 22–28.

[8] Adjuik T. A. , Boi-Dsane N. A. A. , and Kehinde B. A. , Enhancing Dietary Analysis: Using Machine Learning for Food Caloric and Health Risk Assessment, Journal of Food Science. (2024) 89, no. 11, 8006–8021, 10.1111/1750-3841.17421, 39366774. 39366774

[9] Naravane T. and Tagkopoulos I. , Machine Learning Models to Predict Micronutrient Profile in Food After Processing, Current Research in Food Science. (2023) 6, 100500, 10.1016/j.crfs.2023.100500, 37151381. 37151381 PMC10160345

[10] Katidi A. , Xypolitaki K. , Vlassopoulos A. , Kapsokefalou M. , and Kissock K. , Machine Learning Prediction of the Degree of Food Processing, Nature Communications. (2023) 14, no. 1, 10.1038/s41467-023-37457-1. PMC10121643 37085506

[11] Rousseeuw P. J. , Silhouettes: A Graphical Aid to the Interpretation and Validation of Cluster Analysis, Journal of Computational and Applied Mathematics. (1987) 20, 53–65, 10.1016/0377-0427(87)90125-7.

[12] Davies D. L. and Bouldin D. W. , A Cluster Separation Measure, IEEE Transactions on Pattern Analysis and Machine Intelligence. (1979) PAMI-1, no. 2, 224–227, 10.1109/TPAMI.1979.4766909. 21868852

[13] Caliński T. and Harabasz J. , A Dendrite Method for Cluster Analysis, Communications in Statistics. (1974) 3, no. 1, 1–27, 10.1080/03610927408827101.

[14] Arthur D. and Vassilvitskii S. , k-Means++: The Advantages of Careful Seeding, Proceedings of the Eighteenth Annual ACM-SIAM Symposium on Discrete Algorithms, 2007, Society for Industrial and Applied Mathematics, 1027–1035.

